# Comparative *in silico* study of congocidine congeners as potential inhibitors of African swine fever virus

**DOI:** 10.1371/journal.pone.0221175

**Published:** 2019-08-28

**Authors:** Dickson Kinyanyi, Peris Amwayi, Mark Wamalwa, George Obiero

**Affiliations:** 1 Department of Biochemistry and Biotechnology, Technical University of Kenya, Nairobi, Kenya; 2 Department of Biochemistry and Biotechnology, Kenyatta University, Nairobi, Kenya; 3 Center for Biotechnology and Bioinformatics, University of Nairobi, Nairobi, Kenya; University of Calgary, CANADA

## Abstract

African swine fever virus (ASFV) infection is fatal in domesticated pigs, with a mortality rate approaching 100%. This may result in economic losses and threats to food security. Currently, there are no approved vaccines or antiviral therapies for ASFV. Therefore, in this study, we evaluated congocidine congeners and a tris-benzimidazole as potential inhibitors of ASFV transcription using an *in silico* approach. We applied redocking of congocidine and docking of its congeners and a tris-benzimidazole to a receptor containing B-DNA with AT-motifs as a target to mimic conserved ASFV late gene promoters. Subsequently, the binding scores of DNA-ligand docked complexes were evaluated and their binding affinity was estimated. Molecular dynamics (MD) simulation was then used to assess ligand behavior within the minor groove. From our results, it is evident the less toxic congocidine congeners and tris-benzimidazole could dock to AT-rich regions significantly. Additionally, the predicted binding affinities had suitable values comparable to other experimentally determined minor groove binders, MD simulation of the docked DNA-ligand complexes and subsequent molecular trajectory visualization further showed that the ligands remained embedded in the minor groove during the time course of simulation, indicating that these ligands may have potential applications in abrogating ASFV transcription.

## Introduction

DNA is a major target for various types of drugs [1]. Results from the analysis of several high-resolution structures suggest that the minor groove of DNA may function as a receptor for proteins and small molecules [2]. Moreover, drugs that bind to the minor groove may be exploited when pursuing a subset of viruses that replicate in the cytoplasm, such as the African swine fever (ASF) virus (ASFV). ASFV causes ASF, a fatal disease that affects domestic pigs. ASFV infection can affect the food supply, as pork is one of the most commonly consumed kinds of meat worldwide [3].

Currently, there are no vaccines or antiviral drugs approved for use against ASFV [4], and to date, reversible minor groove binders have not been applied or studied in mitigating ASFV replication. A rational study of chemical compounds with potential antiviral activity against ASFV would be extremely useful in identifying prospective prophylactic or therapeutic agents to combat this catastrophic disease or design antivirals. Targeting of viral genomes by antiviral agents may inhibit viral replication and transcription [5–7]. Interestingly, ASFV has conserved AT-rich sequences suggestive of promoter motifs for late gene transcription [8–11], their replacement with equivalent CGCG sequences has been shown to be lethal in stagnating ASFV transcription [8,10]. Therefore, these uniquely conserved AT-rich motifs may serve as a suitable target for DNA minor groove binders in ASFV. Early computational and structural studies on alternating AT elements [12] and binding of ligands netropsin [13], Hoechst 33258 [14,15] and berenil [16] have shown that it is possible to target alternating AT elements of B-DNA minor groove. Significant efforts have been made towards understanding of structural and energetical aspects involved in ligand binding [17,18].

**Fig 1:**
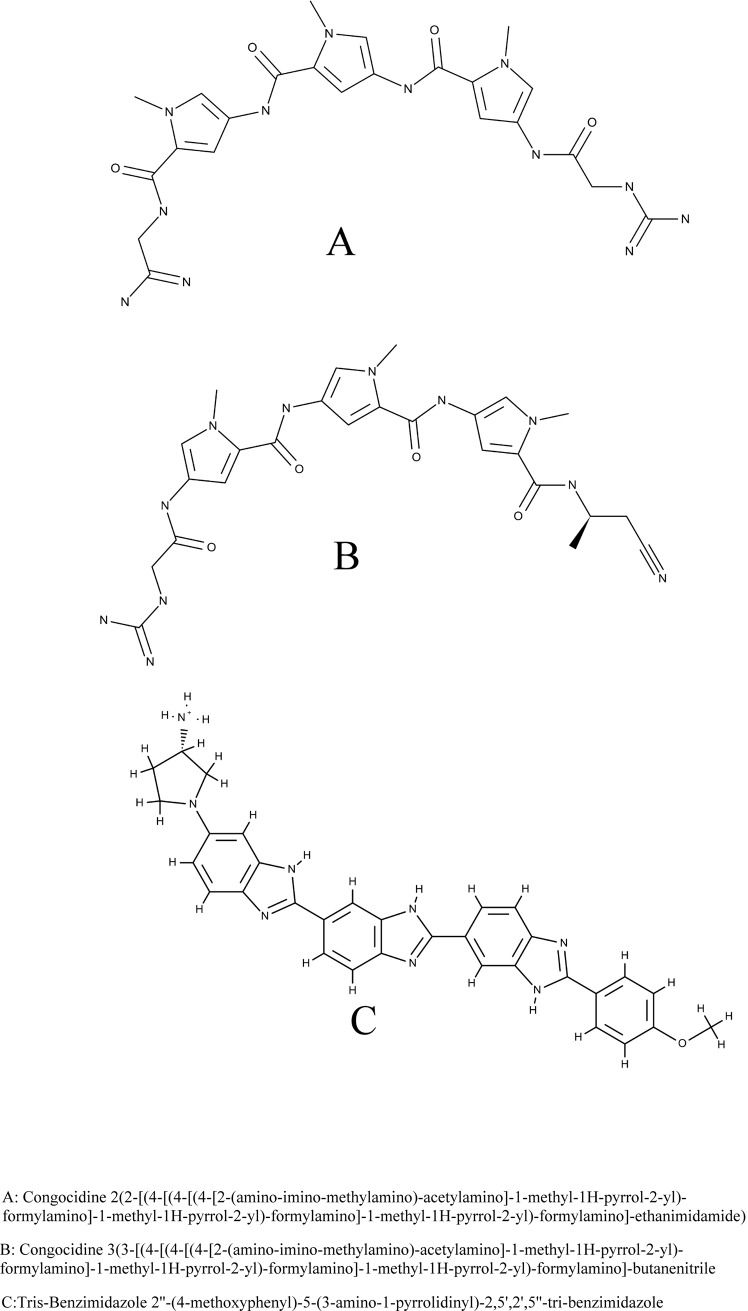
Structures of congocidine congeners and tris-benzimidazole.

Accordingly, in this study, we analyzed the results of docking, binding affinity, and MD simulation of less toxic congocidine congeners and a tris-benzimidazole (Fig 1) to a DNA duplex having multiple combinations of AT motifs observed in core conserved ASFV late promoter regions in an attempt to simulate how these minor groove binders, which have never been used to treat ASF, could be applied as inhibitors for abrogating ASFV late gene transcription.

## Materials and methods

### Docking preparation

The two-dimensional (2D) structures of netropsin/congocidine and tris-benzimidazole were retrieved from PubChem [19] (https://pubchem.ncbi.nlm.nih.gov/), whereas the congeners, congocidine 2 and congocidine 3 were sketched using the Edit molecule utility in the Internal Coordinate Mechanism (ICM) v3.83 algorithm [20]. The congeners were selected based on their documented minimal cytotoxicity [21], whereas the tris-benzimidazole was selected based on its possible bioisosteric ability to bind AT-rich motifs [22]. The starting structure, a netropsin-DNA complex (Protein Data Bank [PDB] ID 473D) [23] having a resolution of 1.58 Å, was retrieved from the PDB [24]. The 473D structure was selected based on the fact that d (CGTATATACG) 2 has the K-mer signature motifs TATATA, ATAT, and TATA, which are similar to conserved AT-rich promoter motifs observed in the ASFV genome that are responsible for late gene transcription in ASFV [8–11]. The presence of nickel atoms observed in the decamer d(CGTATATACG)2 structure has been shown not to introduce any significant distortion in the oligonucleotide structure, whereas the characteristic alternating features of the central AT sequence in the B form of DNA are maintained [23]. The 473D PDB structure imported in ICM was prepared for docking, subsequent binding affinity calculations, and MD simulations by deleting all water molecules, optimizing hydrogen, deleting the unbound terminal guanines, terminal cytosines, terminal nickel atoms and adding the missing heavy atoms and hydrogen (S1 Data). Thereafter, the 473D derivative ligand-receptor complex was converted to an ICM object, and the netropsin ligand was moved from the receptor. The “setup receptor” tool was used to generate a receptor map of the binding site using a grid size of 0.5 Å.

### Redocking and docking of ligands

Semi-flexible docking, which keeps the receptor rigid but the ligand flexible, was performed to predict binding modes of the ligands. The congocidine/netropsin ligand was redocked to the DNA duplexes (S2 Data) to get an estimate of the docking score (S) from the ICM algorithm (S1 Data). Docking of its congeners and the tris-benzimidazole then followed, and the top five scoring values from the hit-list were kept for evaluation. ICM used a Monte Carlo global optimization procedure to predict binding poses for the ligand in the binding pocket [25]. The scoring function (S) in ICM is defined as the sum of energy changes when the ligand binds to the receptor, given as:

$$\Delta S_{\mathrm{bind}}=\Delta E_{\mathrm{IntFF}}+T\Delta S_{\mathrm{Tor}}+\alpha 1\Delta E_{\mathrm{HBond}}+\alpha 2\Delta E_{\mathrm{HBDesol}}+\alpha 3\Delta E_{\mathrm{SolEl}}+\alpha 4\Delta E_{\mathrm{HPhob}}+\alpha {5Q}_{\mathrm{Size}}$$

where Δ *E*_IntFF_ is the change in van der Waals interactions of the ligand and receptor and the internal force-field energy of the ligand, *T*Δ*S*_Tor_ is the change in free energy due to conformational entropy and weighted (α1 − α5), Δ *E*_HBond_ is the hydrogen bond term,

Δ *E*_HBDesol_ accounts for the disruption of hydrogen bonds with solvent, Δ *E*_SolEl_ is the solvation electrostatic energy change upon binding, Δ *E*_HPhob_ is the hydrophobic free energy gain, and *Q*_size_ is the ligand size correction term [26] (http://www.molsoft.com/gui/start-dock.html#interaction-restraints).

The highest scoring predicted docked poses were visualized with ICM Browser, a free downloadable software [27] (V.3.8–5; http://www.molsoft.com/icm_browser.html).

### Binding affinity and free energy prediction

Prior to binding affinity prediction, the ligand-receptor complexes from ICM were minimized using the USCF chimera default protocol [28]. Preddicta (http://www.scfbio-iitd.res.in/software/drugdesign/preddicta.jsp), an all-atom energy-based computational protocol, was used to approximate DNA-ligand binding affinity [29]. Calculated binding energies (cbe) from preddicta have been shown to have high correlation coefficients of 0.95 (R^2^ = 0.90) and 0.96 (R^2^ = 0.93), using linear regression plots against experimental binding free energies (ΔG°) and change in thermal melting temperature ΔT_m_ respectively [29]. The energy function used for the calculated DNA-ligand binding energy was represented by the equation below:

$$\Delta {G{}^{\circ}}_{\mathrm{cbe}}=\Delta {H{}^{\circ}}_{\mathrm{el}}+\Delta {H{}^{\circ}}_{\mathrm{vdw}}‐T\Delta {S{}^{\circ}}_{\mathrm{rt}}+\Delta {G{}^{\circ}}_{w}$$

where G° _cbe_ is the calculated binding energy, H° _el_ is the electrostatic term, ΔH° _vdw_ is the van der Waals term, TΔS°_rt_ represents the rotational and translational entropy changes on complex formation, and ΔG°_w_ is a hydration term. The overall ΔG° _cbe_ was used to predict ΔT_m_ and ΔG° for docked complexes using the equations below:

$$\Delta {G{}^{\circ}}_{\mathrm{pred}}(\mathrm{predicted})=(\Delta {G{}^{\circ}}_{\mathrm{cbe}}‐62.215)/7.909$$

$$\Delta T_{m}(\mathrm{predicted})=(\Delta {G{}^{\circ}}_{\mathrm{cbe}}‐2.438)/(‐1.468)$$

### Molecular dynamics (MD) simulation

To study the behavior of the docked ligand within the minor groove, the top scoring docked poses in PDB format from congocidine congeners and the tris-benzimidazoles (S3 Data) were exported from ICM version 3.83 to Desmond version 5.3 [30] for MD simulation, prior to performing the MD simulation. The DNA-ligand docked complexes were preprocessed using Prepwizard tool. A solvation model of a Monte-Carlo-equilibrated, transferable intermolecular potential three-point (TIP3P) water bath was used, with box shape orthorhombic boundary conditions, buffer box size calculation method, distances of 10 Å × 10 Å × 10 Å, and a minimized box volume. For DNA-congocidine 2, DNA-congocidine 3, and DNA-tris-benzimidazole docked complexes, 14 Na^+^, 15 Na^+^, and 15 Na^+^ ions, respectively, were used for neutralization. Finally, after these initial equilibration conditions, MD simulation was performed by applying the OPLS3 force field, and the MD simulations were run for 5 ns at a default temperature of 300 K. During the MD simulation, intermediate structures were saved at a time interval of 10 ps and were superimposed with their native structures to deduce the root mean squared deviation (RMSD) of the ligand. The MD trajectories were visualized, animations were rendered, and 2D ligand interaction diagrams were systematically sampled at intervals of 100 frames to analyze the ligand-receptor complex behavior in the system.

### SwissADME prediction

*In silico* drug-related properties like LogP and solubility were carried out by SwissADME [31] (http://www.swissadme.ch/), an online server that predicts Absorption Distribution Metabolism Excretion (ADME) parameters.

## Results

### Docking and redocking results

**Fig 2:**
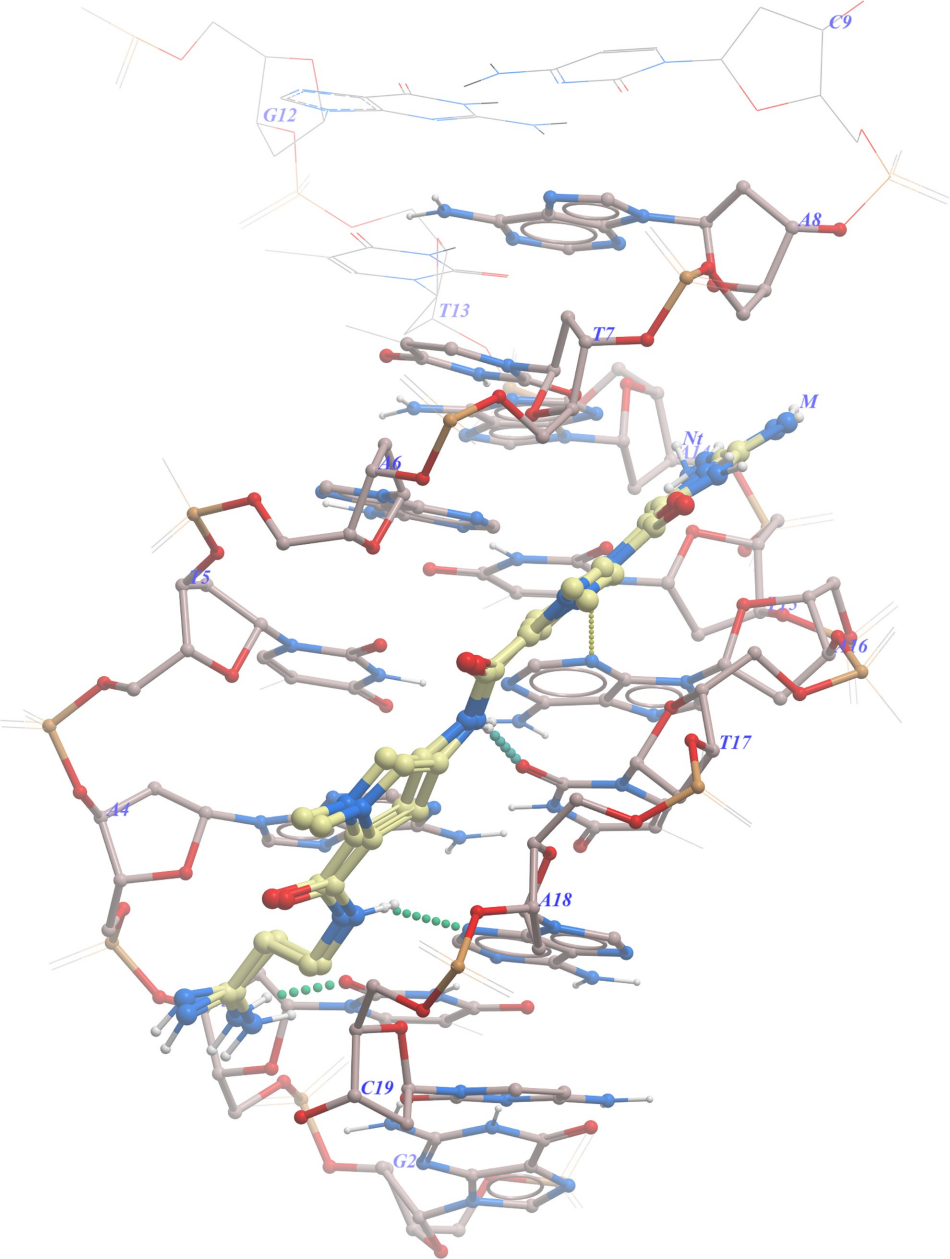
A similar predicted docking pose of congocidine versus the 473D derivative. The atoms form hydrogen bonds (in dotted spheres) with acceptor atoms N3 of adenine and O2 of thymine.

The binding score (S) from congocidine /netropsin redocking to the minor groove was found to be approximately (-43.35), with an RMSD to the 473D native derivative of 0.22 Å (Fig 2 and S1 Data). This (S) score may serve as a critical value in determining an approximation of the expected docking score for the tested minor groove binders used in this study.

To establish if the congocidine congeners and the tris-benzimidazole derivative had significant binding ability to the 5′ (GTATATAC) 2 duplex, congocidine congeners and tris-benzimidazole were docked to the DNA duplex (Table 1 and S2 Data). The top five stacked conformer poses from the docked complexes were found to have a range of -47.22 to -57.83 for congocidine 2, -39.40 to -47.27 for congocidine 3, and -42.56 to -50.29 for the tris-benzimidazole. For the tested minor groove binders in this study, the score range was comparable to those observed in congocidine -39.37 to -43.35. A redocking replicate of congocidine using the 5′ (GCTATATACG) 2 duplex showed the score was within the range of -39.37 to -43.35 (S2 Data). Moreover, based on a benchmarked analysis of the ICM algorithm, a score of -32 or lower is considered significant (http://www.molsoft.com/icmpro/faq-docking.html#faq-score). This score has been used in various studies to discriminate binders from non-binders, with more negative scores representing more likely binding interactions and higher binding affinity of a particular ligand [32,33]. From the stacked conformers in this study, all of the top five docked poses for minor groove binders had the score (S) less than -32, strongly suggesting that both the congocidine congeners and tris-benzimidazole could significantly bind to the TATATA motifs like congocidine.

**Table 1:** ICM top 5 docking parameters for minor groove binders.

NAME	Score^a^	Hbond^b^	Hphob^c^	VwInt^d^	Eintl^e^	Dsolv^f^	SolEl^g^
**Congocidine**	-43.35	-8.10	-6.10	-47.68	6.01	27.50	7.10
**Congocidine**	-40.71	-10.16	-6.23	-46.91	9.19	33.50	11.14
**Congocidine**	-40.61	-9.32	-6.33	-43.51	11.18	29.09	7.70
**Congocidine**	-40.02	-11.34	-6.10	-43.55	14.28	34.48	10.02
**Congocidine**	-39.37	-9.30	-5.51	-40.38	16.07	29.48	6.82
**Congocidine 2**	-57.83	-13.44	-7.41	-56.33	13.60	35.23	12.21
**Congocidine 2**	-54.94	-12.76	-7.55	-55.49	12.91	35.26	12.81
**Congocidine 2**	-48.21	-10.12	-7.47	-51.98	7.90	33.70	9.24
**Congocidine 2**	-47.48	-11.04	-7.64	-55.70	12.07	37.30	15.61
**Congocidine 2**	-47.22	-10.22	-7.43	-51.74	8.96	33.17	11.33
**Congocidine 3**	-47.27	-9.16	-8.42	-54.42	12.85	32.34	13.21
**Congocidine 3**	-46.70	-8.56	-8.65	-55.09	11.91	33.47	11.75
**Congocidine 3**	-44.83	-8.12	-8.15	-53.65	10.80	33.95	9.65
**Congocidine 3**	-41.47	-6.82	-7.97	-49.21	11.03	28.53	9.01
**Congocidine 3**	-39.40	-7.32	-7.96	-50.34	8.61	31.12	12.83
**Tris-benzimidazole**	-50.29	-8.45	-9.02	-53.62	10.42	29.34	13.28
**Tris-benzimidazole**	-47.84	-9.30	-8.67	-48.51	9.55	29.29	12.18
**Tris-benzimidazole**	-44.08	-5.89	-9.16	-54.48	11.35	31.60	11.72
**Tris-benzimidazole**	-42.58	-5.20	-9.09	-53.13	5.51	29.21	11.99
**Tris-benzimidazole**	-42.56	-6.27	-7.98	-47.09	5.00	25.52	10.03

^a^Score is the ICM score (-32 and lower are generally considered good scores)

^b^Hbond is hydrogen bond energy (lower values are better)

^c^Hphob is the hydrophobic energy of the surface exposed to water (lower values are better)

^d^VwInt is the van der Waals interaction energy (lower values are better)

^e^Eintl is the internal conformation energy of the ligand (lower values are better)

^f^Dsolv is the desolvation of exposed h-bond donors and acceptors (lower values are better)

^g^SolEl is the solvation electrostatic energy change upon binding (lower values are better)

**Fig 3:**
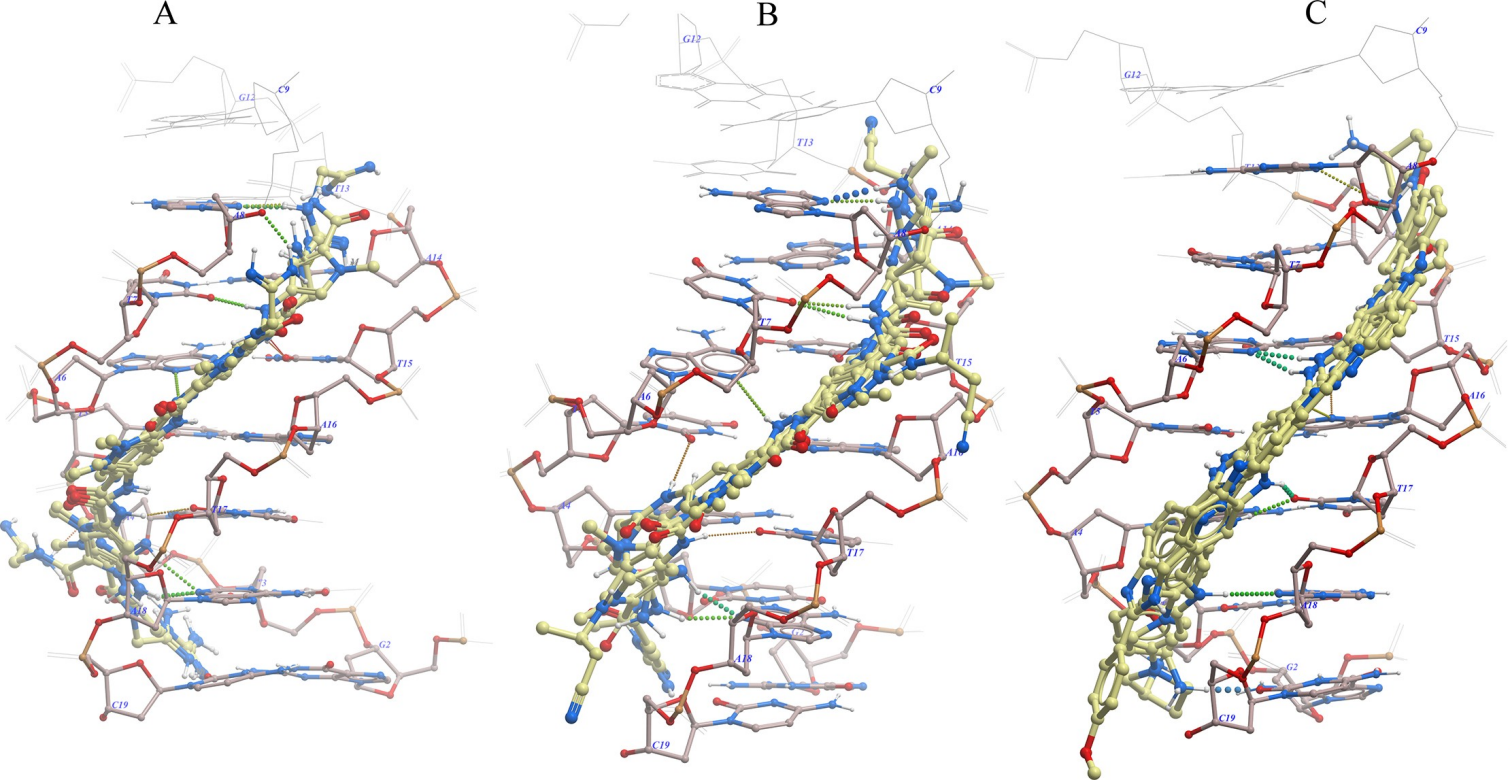
**Minor groove binders docking to the central d (GTATATAC) 2:** (A) congocidine 2, (B) congocidine 3, and (C) tris-benzimidazole (hydrogen bonds are in dotted spheres).

From the docked poses of congocidine 2, congocidine 3, and tris-benzimidazole, the complete coverage of the TATATA region was observed in both forward and reverse orientation of the ligand to the self-complementary sequence 5′ (GTATATAC) 2, a familiar binding pattern characterized by hydrogen bonding of the amide nitrogen NH to acceptor atoms of N3 adenine (A), and O2 to thymine (T) was observed for the sampled top five docked conformer poses of congocidine congeners to the central 5′ TATATA (Fig 3A and 3B, S2 Data). A similar binding pattern was also observed for the tris-benzimidazole top five conformers docked to the central TATATA. The inner facing NH groups of the benzimidazole subunits of the tris-benzimidazole participated in hydrogen bonding with acceptor atoms of N3 adenine (A), and O2 to thymine (T) bases of the duplex 5′ (GTATATAC)2 DNA, in a manner analogous to that observed in congocidine and its congeners (Fig 3C, S2 Data), showing that these minor groove binders could function as bioisosteres of each other, to some extent.

Additionally, from the top five sampled stacked conformers of congocidine congeners, the possibility of hydrogen bonding between terminal NH groups and the O atoms of the DNA strands was observed. This may have resulted from the increased degrees of freedom of the flexible terminal NH groups that brought the groups within the hydrogen bonding distance (Fig 3A and S2 Data). A similar observation was made involving the flexible 3-amino-1-pyrrolidinyl group at the terminal of the tris-benzimidazole, where a possibility of hydrogen bonding with the O4′ atom of the DNA strands and O2′ of cytosine 19 was observed (Fig 3C and S2 Data).

For all minor groove binders, the crescent-shaped curvature of ligands was complementary to the floor of the minor groove and spanned the entire 5′ TATATA of the minor groove (Fig 3 and S2 Data). A combination of shape complementarity and the associated favorable van der Waals contribution of the minor groove has been described as one of the decisive factors in the drug binding process [34,35].

From the docking score parameters of minor groove binders with DNA, it was evident that the greatest contributing factor from the ICM scoring function came from van der Waals interaction energy (Table 1). Higher values were observed from the tris-benzimidazole and congocidine congeners, in comparison with congocidine. This may be attributed to the larger binding length from the extra N-methylpyrrole and benzimidazole rings along the minor groove, in comparison with congocidine. Generally, close van der Waal contacts between sandwiching sugar ring-phosphate backbone chains and unsaturated π electrons of the N-methylpyrrole ring in minor groove binders have been shown to play a major role in the stability of the DNA-ligand complexes [36], a similar stabilizing interplay of van der Waal interaction would be manifested in the tris-benzimidazole, where interactions involving the O4′ oxygens of the ribose rings are in contact with the unsaturated π electrons of the benzimidazole subunits [14].

### Binding affinity and free energy prediction results

The DNA-ligand binding affinity calculations for the docked complexes of congocidine congeners and the tris-benzimidazole were carried out using preddicta algorithm [29]. Theoretically calculated binding affinities of the sampled top five minimized conformers complexes are summarized in Table 2. For the docked complexes of the reversible minor groove binders (S4 Data), the calculated binding affinities were within the linear interpolation range of the known benchmarked experimental values in the preddicta data set [29]. The calculated total binding energies and binding affinities of the complexes had negative values, reflecting that the drug-DNA interaction was feasible for docked complexes of congocidine congeners and tris-benzimidazole. The ranking order for the binding was again dominated by van der Waal factors, with the longer congocidine congeners and tris-benzimidazole having higher values than congocidine. These results further prove that congocidine congeners and tris-benzimidazole have a binding affinity that is better than congocidine (Table 2**).**

**Table 2:** Binding affinity and free energy prediction of minor groove binders.

Ligand	ICM-Score	Total Electrostatics ΔH°_el_ (kcal/mol)	Total van der Waals ΔH° _vdw_ (kcal/mol)	Rotational Translational Entropy TΔS°_rt_ (kcal/mol)	Hydration Free Energy ΔG°_w_ (kcal/mol)	Total energy ΔG° _cbe_ (kcal/mol)	Predicted Delta ΔTm(K)	Predicted Binding Affinity ΔG °_pred_ (kcal/mol)
**Netropsin(473D)**		-5.6	-26.6	25.5	-11.8	-18.6	14.3	-10.2
**Congocidine redock**	-43.35	-5.0	-27.9	25.5	-12.0	-19.5	14.9	-10.3
**Congocidine 2**	-57.83	-6.2	-33.5	26.0	-15.2	-28.7	21.2	-11.5
**Congocidine 2**	-54.94	-6.4	-33.6	26.0	-15.9	-29.7	21.9	-11.6
**Congocidine 2**	-48.21	-7.8	-30.3	26.0	-15.2	-27.3	20.3	-11.3
**Congocidine 2**	-47.48	-5.9	-34.5	26.1	-15.3	-29.6	21.8	-11.6
**Congocidine 2**	-47.22	-5.9	-31.2	26.0	-15.2	-26.3	19.6	-11.2
**Congocidine 3**	-47.27	-5.5	-35.0	26.1	-17.9	-32.3	23.7	-12.0
**Congocidine 3**	-46.70	-5.6	-33.7	26.1	-18.3	-31.4	23.1	-11.8
**Congocidine 3**	-44.83	-5.8	-34.2	26.1	-17.0	-30.9	22.7	-11.8
**Congocidine 3**	-41.47	-4.6	-30.2	26.0	-17.2	-25.5	19.4	-11.2
**Congocidine 3**	-39.40	-4.6	-30.1	26.0	-16.9	-25.5	19	-11.1
**Tris-benzimidazole**	-50.29	-3.4	-37.9	26.1	-19.9	-35.2	25.6	-12.3
**Tris-benzimidazole**	-47.84	-3.5	-29.5	26.1	-18.0	-24.9	18.6	-11.0
**Tris-benzimidazole**	-44.08	-3.1	-34.3	26.1	-19.7	-31	22.8	-11.8
**Tris-benzimidazole**	-42.58	-4.2	-33.9	26.1	-18.9	-30.9	22.7	-11.8
**Tris-benzimidazole**	-42.56	-2.4	-30.7	26.0	-17.3	-24.4	18.3	-10.9

### MD simulation results

#### Congocidine 2

**Fig 4:**
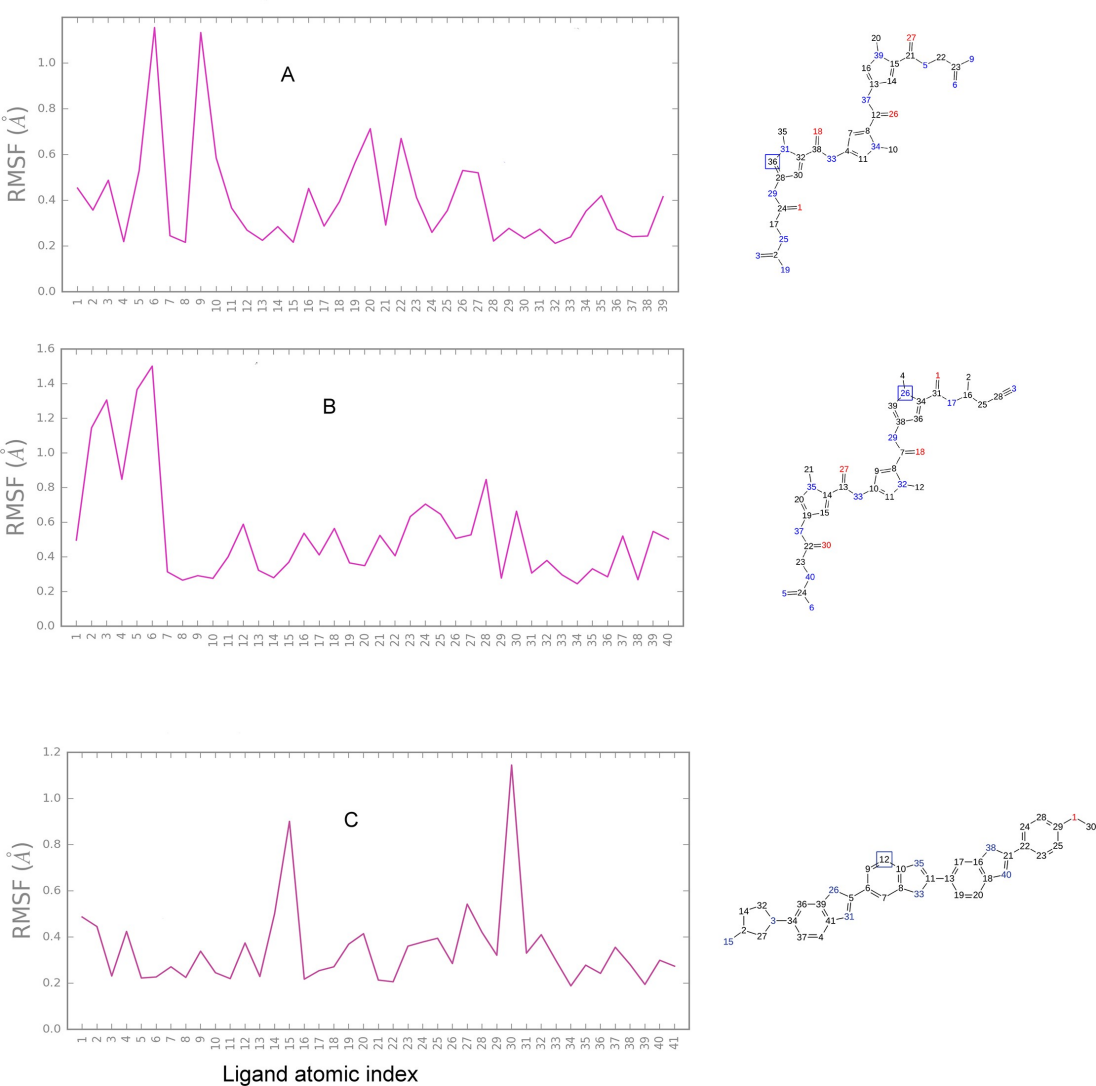
Time-dependent root means square fluctuation (RMSF) plot in Angstroms for minor groove binders showing the heavy atom fluctuations of the ligand within the minor groove.

The assessment of ligand behavior within the minor groove binding pocket of the DNA-congocidine 2 complex showed the heavy atoms 6 and 9 of the terminal guanidinium portion had the highest root mean square fluctuation (RMSF) (Fig 4A), probably because the NH_2_ amino group of the guanine base in the minor groove exerted steric hindrance to the entry of the ligand amino group into the minor groove. The least amount of fluctuation was observed in heavy atoms 29, 33, 37, buried in the minor groove. From the 2 dimensional ligand extracts (S5 Data), DNA-congocidine 2 interaction patterns were characterized by hydrogen bonding of amide nitrogen NH to acceptor atoms of N3 adenine and O2 to thymine, with both strands of the DNA duplex 5′ (GTATATAC)2, while pi-cationic bonds were observed to exist between both guanidinium and ethanimidamide amino groups and terminal adenine and thymine bases. Water molecules were also observed to bridge terminal hydrogen bonds involving adenine, thymine, and cytosine during the MD simulation within a range cutoff of 4 Å (S5 Data).

**Fig 5:**
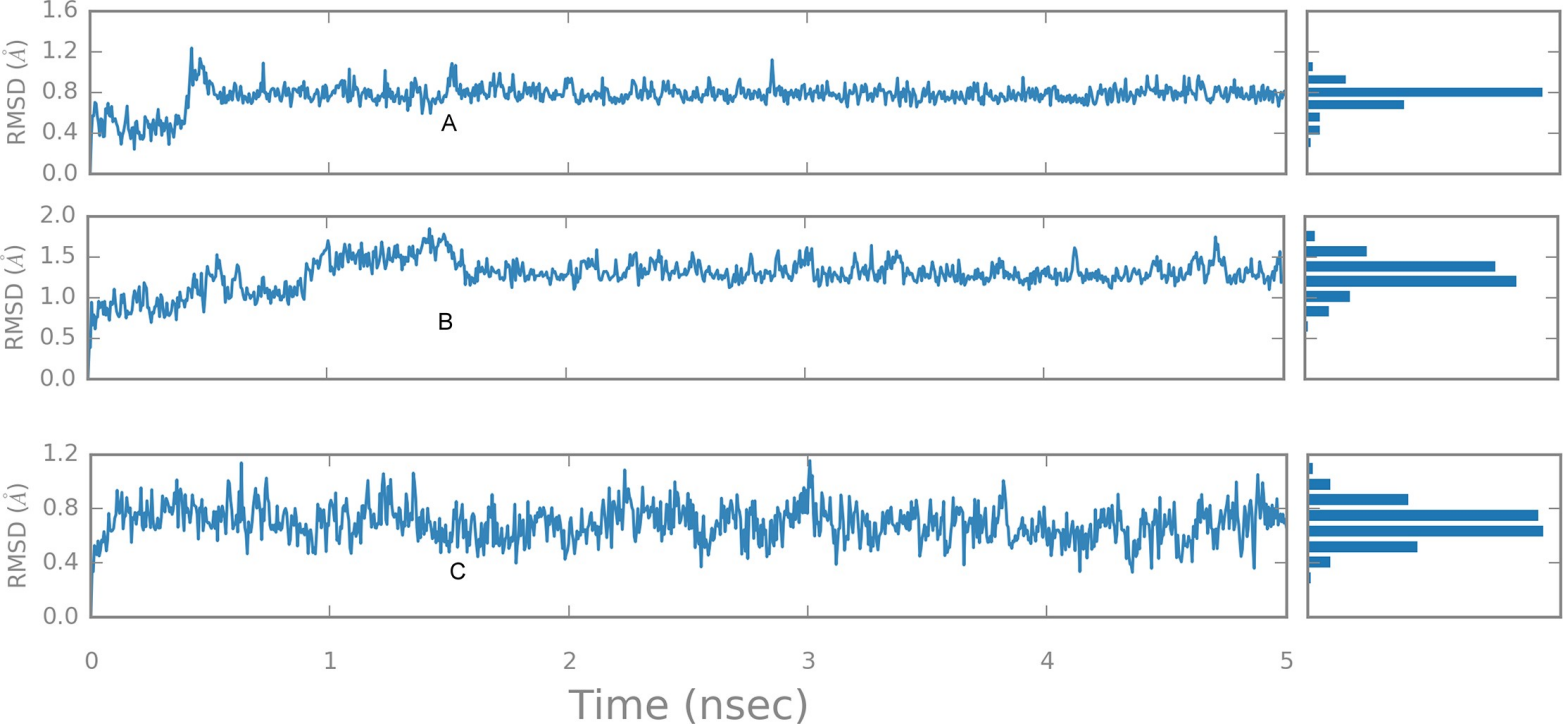
**RMSD of the average ligand structure as a function of time and stability profiles for the MD simulation:** (A) congocidine 2, (B) congocidine 3, and (C) tris-benzimidazole.

Overall, congocidine 2 RMSD remained at approximately 0.8 Å, after the transition from reference conformation at time t = 0. Deviations around 0.8 Å had a minimal spread around the mode after 0.5 ns and seemed to have stabilized (Fig 5A). Molecular trajectory visualization of simulated animation (S6 Data) showed that the ligand remained bound in the minor groove during the time course of the simulation.

#### Congocidine 3

In the RMSF analysis of congocidine 3, the least amount of RMSF was observed in heavy atoms, deep in the less solvent exposed part of the minor groove (heavy atoms 29, 33, 17, 37) (Fig 4B). The heavy atoms 29 and 33 had less space to move around, and thus, a lower RMSF of approximately 0.2 Å deviation was observed in this region.

Additionally, the terminal butanenitrile and guanidinium portion of congocidine 3, which are solvent exposed, were found to show high root mean square fluctuations (RMSFs), probably due to steric hindrance from C2 amino group of the guanine base towards the guanidinium amino groups and the increased degrees of freedom of movement of rotatable bonds towards the solvent-exposed terminal heavy atoms (Fig 4B). A maximal atomic RMSF of 1.5 Å and 1.4 Å were observed for the heavy atoms 6 and 5 respectively of the guanidinium portion, while a 1.3 Å RMSF was observed for heavy atom 3 of the butanenitrile portion.

Most of the ligand fragments remained within the minor groove, as visualized by animation (S7 Data). The sampled 2-dimensional ligand extraction showed that interactions of DNA-congocidine 3 were characterized by hydrogen bonding of amide nitrogen (NH) to acceptor atoms of N3 adenine (A) and O2 to thymine (T). Moreover, the terminal guanidinium end was also observed to be capable of hydrogen bonding with adenine (A4), water molecules formed bridging interactions with adenine and thymine bases at the floor of the minor groove (S5 Data).

Overall ligand RMSD was centered around 1.2 Å, relative to the reference conformation at time t = 0 and seemed to have stabilized after 1.5 nanoseconds (Fig 5B).

#### Tris-benzimidazole

The terminal portions of tris-benzimidazole, which are solvent exposed, showed high RMSFs, probably due to the increased degree of freedom of the solvent-exposed terminals (Fig 4C). A maximal atomic RMSF of approximately 1.1 Å, involving heavy atom 30 was observed at the methoxyphenyl end while the 3 amino-1-pyrrolidinyl end had a maximal RMSF of 0.8 Å. The least amount of fluctuation was observed in heavy atoms deep in the narrow and less solvent exposed part of the minor groove (heavy atoms 26, 33, 38) (Fig 4C). These bonds probably had less space to move around and were buried within the minor groove, thus, a lower RMSF of slightly above 0.2 Å deviation was observed in these regions. The ligand fragment remained within the minor groove, as visualized by the animation (S8 Data). The sampled 2-dimensional ligand extraction patterns (S5 Data) showed that interactions of DNA-tris-benzimidazole had the inner facing nitrogen atom of the benzimidazole subunits of the tris-benzimidazole, participating in hydrogen bonding with acceptor atoms of N3 adenine (A) and O2 to thymine (T) bases of the duplex 5′ (GTATATAC)2 DNA. Moreover, the terminal 3 amino-1-pyrrolidinyl end was also observed to be capable of hydrogen bonding with adenine, thymine and forming a pi-cation bond with cytosine. Water molecules were involved in forming bridging interactions with adenine and thymine at the floor of the minor groove (S5 Data). Overall ligand RMSD was centered between 0.5 Å and 0.6 Å, relative to the start frame (Fig 5C).

### LogP evaluation

Lead potential evaluation for congocidine 2, congocidine 3, and the tris-benzimidazole by SwissADME revealed low LogP values of -1.53 for congocidine 2 and -0.67 for congocidine 3; these congeners were very soluble (Table 3). The property of solubility for these congeners could be advantageous in parenteral usage, as a drug has to be highly soluble in water to deliver a sufficient quantity of active ingredient in a small volume of pharmaceutical dosage [37]. The tris-benzimidazole predicted lead potential, revealed a higher predicted LogP (lipophilicity) value of 3.45 and poor solubility in water, nonetheless, having a large LogP value has been largely been associated with efficient microencapsulation or formation of liposomes [38]. Liposomes are a widely used successful system when targeting macrophages [39] and macrophages are the primary site for ASFV infection [40]. As such, tris-benzimidazole packaged in liposomes may potentially be engineered to show significant accumulation in macrophages and to minimize potential toxic effects [41,42].

**Table 3:** ADME properties of select minor groove binders.

Compound	Log P	Solubility
**Congocidine 1**	-1.59	Very Soluble
**Congocidine 2**	-1.53	Very soluble
**Congocidine 3**	-0.67	Very soluble
**Tris-benzimidazole**	3.45	Poorly Soluble
**Hoechst 33258**	2.56	Moderately soluble

## Discussion

As an antiviral, congocidine has been shown to inhibit the multiplication of viruses, such as Vaccinia virus [43], Shope fibroma virus [43], and Herpes simplex virus [21]. However, unlike the parent drug congocidine, congocidine tri pyrrole derivatives have been shown to display more potency and less cytotoxicity compared to congocidine and distamycin A [21], making them suitable ligands for targeting the ASFV viral genome, moreover, ASFV post-replicative genes are 80% AT-rich and display apparent conserved sequence similarity in its late gene promoters [10].

From docking and subsequent simulation results of congocidine 2, congocidine 3, and tris-benzimidazole, it was evident that these minor groove binders could significantly dock with the AT elements in the minor groove of duplex d (GTATATAC) 2 with coverage spanning the entire 5′ TATATA sequence. Therefore, a model involving the interaction of minor groove binders studied herein and ASFV late viral promoters may be postulated in the potential inhibition of ASFV late gene transcription. We postulate late gene transcription because it occurs in the cytoplasm, after viral genome replication, and thus the viral genome is accessible to solutes [44]. To further highlight with an example, the B646L gene encoding the major capsid protein p72, has an indispensable 5′ TATATA motif in its core promoter region [8–11]. In our docking and simulation experiment, significant binding to a central 5′ TATATA motif was demonstrated using congocidine congeners and tris-benzimidazole, with significant scores in ICM and good binding affinity using preddicta. Thus, it may be foreseen that congocidine congeners and tris-benzimidazoles have the potential to bind conserved 5′ TATATA promoter motifs including B646L[8], A224L[45], B438L[46], C129R, E165R[47] and I329L[10], or at least conserved AT-rich late promoter 4-mer motifs (S4 Data) in ASFV, and may thereby affect the transcription of multiple ASFV late genes via steric interference, involving melting and unwinding of DNA or interfere with transcription factors that rely on direct or indirect anchorage to AT-rich motifs. One such transcription factor, pB263R, having TATA-binding protein-like features, has been predicted to exist in ASFV [48]. TATA-binding protein (TBP) typically binds to TATA motifs and is a primary anchor of other transcription factors involved in binding to DNA [9].

Consistent with our postulated ASFV TBP/DNA inhibition by congocidine congeners and the tris-benzimidazole in ASFV, studies have shown that Vaccinia virus, a nucleocytoplasmic large DNA virus, has intermediate and late promoter elements that are targeted by TBP [49]. Moreover, intermediate and late gene transcription processes in Vaccinia virus are inhibited by the minor groove binders bisbenzimidazole [7] and distamycin A [49,50]. It turns out that Vaccinia virus has indispensable conserved late TAAAT(G/A) and intermediate TAAAT promoters [7, 8] while ASFV has indispensable conserved late TATA-like promoter motifs ATAT, TATA, ATAA, and TATATA [9,10]. These motifs are consistent with the binding requirements of MGBs and TBPs. In addition, evaluations by gel mobility shift assays have shown that minor groove binders are effective inhibitors of DNA/TBP interactions [51], leading to transcription stagnation [49–52]. The likelihood of ASFV inhibition through transcription stagnation involving AT-rich late promoters is thereby somewhat predictable, using both congeners and tris-benzimidazoles.

## Conclusion

In this study, we examined how less toxic congocidine congeners and a tris-benzimidazole bioisostere could be used for targeting of ASFV temporal transcription processes. The results of this *in silico* study showed, for the first time, how minor groove binders could be used in targeting conserved late gene AT motifs in ASFV that are important for transcription. While our findings are largely *in silico* and provide a model for understanding, explaining and exploitation of the potential inhibition of ASFV transcription, future experimental verification, both *in vivo* and *in vitro*, is necessary.

## Supporting information

S1 Data ICM congocidine redocked. (ZIP)

S2 Data ICM files Congo 2, Congo3, Tribz. (ZIP)

S3 Data Simulation input PDBs. (ZIP)

S4 Data Minimized PDB for predictta. (ZIP)

S5 Data 2D ligand extract files. (ZIP)

S6 Data Congocidine 2 simulated animation. (ZIP)

S7 Data Congocidine 3 simulated animation. (ZIP)

S8 Data Tris-benzimidazole simulated animation. (ZIP)
