# Supplementary material for: Comparative in silico study of congocidine congeners as potential inhibitors of African swine fever virus
Source: PLoS One. 2019 Aug 28;14(8):e0221175. doi: 10.1371/journal.pone.0221175 (PMC6713398; doi:10.1371/journal.pone.0221175)
Supplement: S2 Data — (ZIP) [file pone.0221175.s002.zip › S2 Data ICM files Congo 2,Congo3,Tribz/S2 figure.pdf]

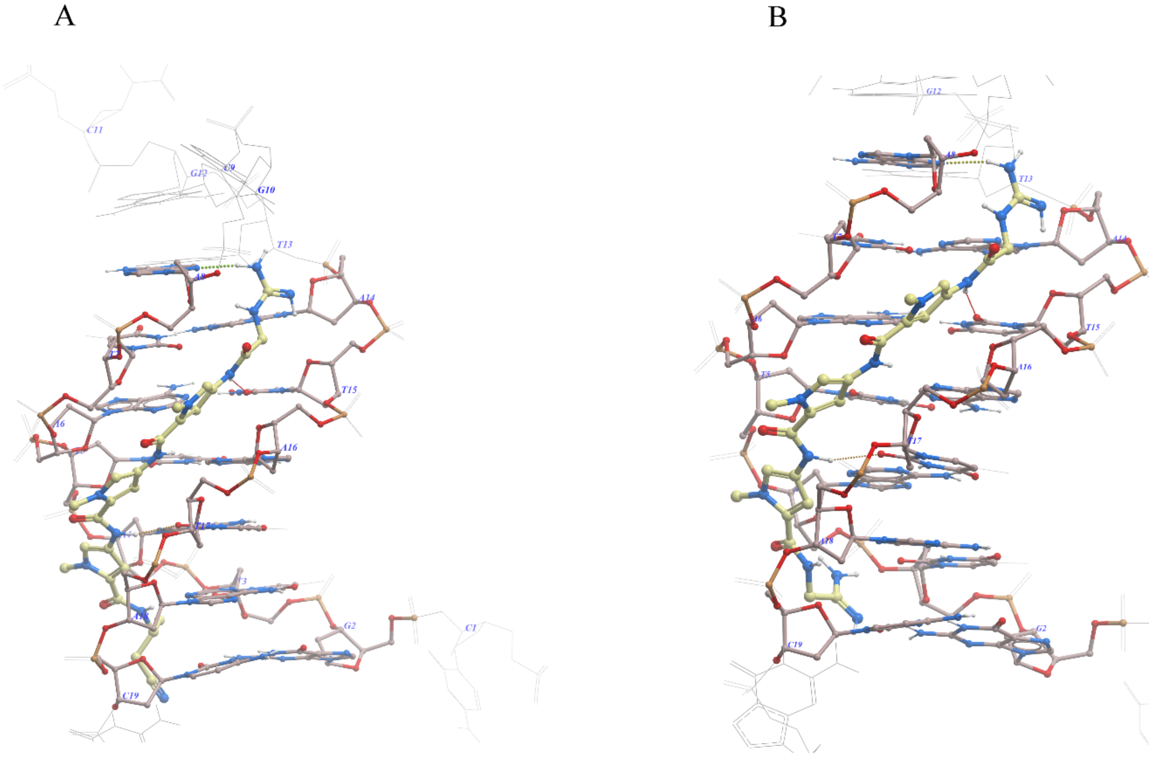

Top scoring nearly similar predicted docking poses of (A) congocidine 3 and (B) congocidine 2 curved along the minor groove of the DNA duplex d(GCTATATACG)2. The structures have similarity to those observed in earlier studies of (Gresh and Pullman, Theoret. Chim. Acta, 1984, 64, 383) and (Gresh and Pullman, Mol. Pharm. 1984, 25, 452)

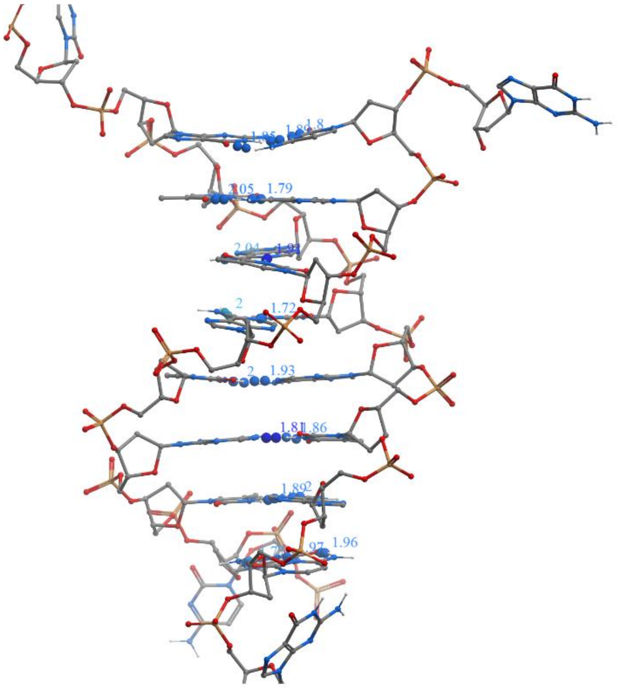

A

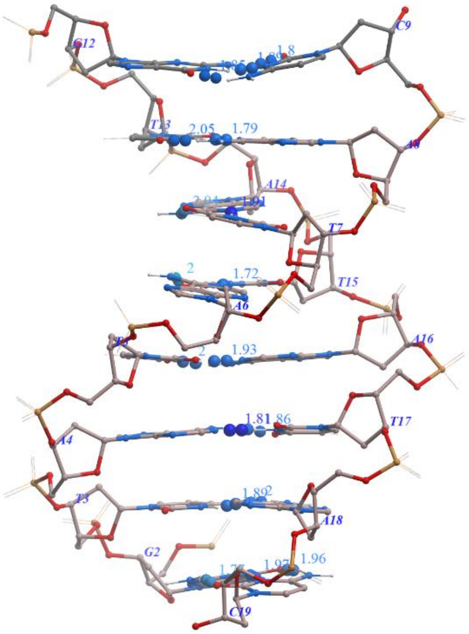

B

Dimensions of (A) untruncated d(CGTATATACG)2 and (B) truncated d(CGTATATACG)2 hydrogen bond distances, the hydrogen bonding distances are identical for bonded AT/TA and CG/GC pairs.
