# Supplementary material for: Comparative in silico study of congocidine congeners as potential inhibitors of African swine fever virus
Source: PLoS One. 2019 Aug 28;14(8):e0221175. doi: 10.1371/journal.pone.0221175 (PMC6713398; doi:10.1371/journal.pone.0221175)
Supplement: S4 Data — (ZIP) [file pone.0221175.s004.zip › S4 Data Minimized PDB for predictta/S4 Table.docx]

**S4 Table: Binding affinity and free energy prediction of minor groove binders to the duplex d( CGCGATATCGCG)2.**

| **Ligand** | **Total**  **Electrostatics**  **ΔH°_el_**  **(kcal/mol)** | **Total van**  **der Waals**  **ΔH° _vdw_**  **(kcal/mol)** | **Rotational**  **Translational**  **Entropy**  **TΔS°_rt_**  **(kcal/mol)** | **Hydration**  **Free**  **Energy**  **ΔG°_w_**  **(kcal/mol)** | **Total energy**  **ΔG° _cbe_**  **(kcal/mol)** | **Predicted Delta ΔTm(K)** | **Predicted Binding**  **Affinity**  **ΔG °_pred_**  **(kcal/mol)** |
| --- | --- | --- | --- | --- | --- | --- | --- |
| **1vtj** | -2.9 | -28.7 | 25.5 | -12.7 | -18.8 | 14.4 | -10.2 |
| **Congocidine 2 p1** | -4.9 | -36.9 | 26.1 | -14.1 | -29.8 | 22.0 | -11.6 |
| **Congocidine 2 p2** | -6.6 | -38.3 | 26.1 | -15.7 | -34.4 | 25.1 | -12.2 |
| **Congocidine 2 p3** | -4.2 | -37.7 | 26.1 | -14.9 | -30.7 | 22.6 | -11.6 |
| **Congocidine 3 p1** | -4.3 | -35.6 | 26.0 | -16.6 | -30.5 | 22.4 | -11.7 |
| **Congocidine 3 p2** | -3.2 | -29.8 | 26.1 | -17.3 | -24.2 | 18.1 | -10.9 |
| **Congocidine 3 p3** | -4.2 | -36.3 | 26.1 | -17.1 | -31.5 | 23.1 | -11.8 |
| **Tris-benzimidazole p1** | -3.6 | -39.0 | 26.1 | -19.8 | -36.2 | 26.3 | -12.4 |
| **Tris-benzimidazole p2** | -3.4 | -40.6 | 26.2 | -20.1 | -37.9 | 27.5 | -12.7 |
| **Tris-benzimidazole p3** | -3.7 | -36.6 | 26.0 | -18.3 | -32.6 | 23.9 | -12.0 |
