# Supplementary material for: Comparative in silico study of congocidine congeners as potential inhibitors of African swine fever virus
Source: PLoS One. 2019 Aug 28;14(8):e0221175. doi: 10.1371/journal.pone.0221175 (PMC6713398; doi:10.1371/journal.pone.0221175)
Supplement: S5 Data — (ZIP) [file pone.0221175.s005.zip › congocidine 3/desmond_md_job_18-out_pl_1.pdf]

Simulation Interactions Diagram Report

Simulation Details

Jobname: desmond_md_job_18
Entry title: desmond_setup_27_3-out

CPU #	Job Type	Ensemble	Temp. [K]	Sim. Time [ns]	# Atoms	# Waters	Charge
1	mdsim	NPT	300.0	5.008	7851	2412	0

Ligand Information

SMILES	N#CC[C@@H](C)NC(=O)c1cc(cn1C)NC(=O)c2cc(cn2C)NC(=O)c3cc(cn3C)NC(=O) CNC(N)=[NH2+]
PDB Name	'RES'
Num. of Atoms	72 (total) 40 (heavy)
Atomic Mass	550.605 au
Charge	+1
Mol. Formula	C25H32N11O4
Num. of Fragments	10
Num. of Rot. Bonds	16

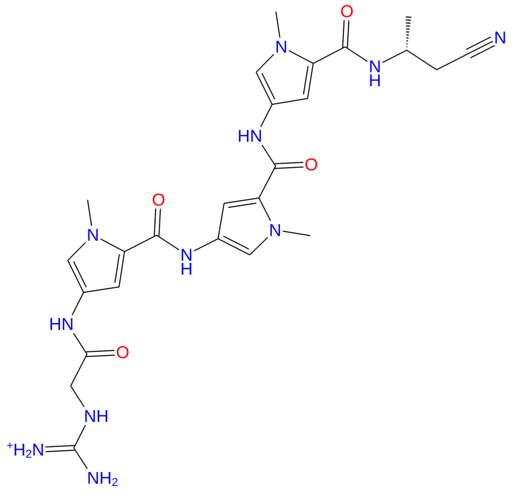

Counter Ion/Salt Information

Type	Num.	Concentration [mM]	Total Charge
Na	22	165.837	+22
Cl	7	52.766	-7

Ligand RMSF

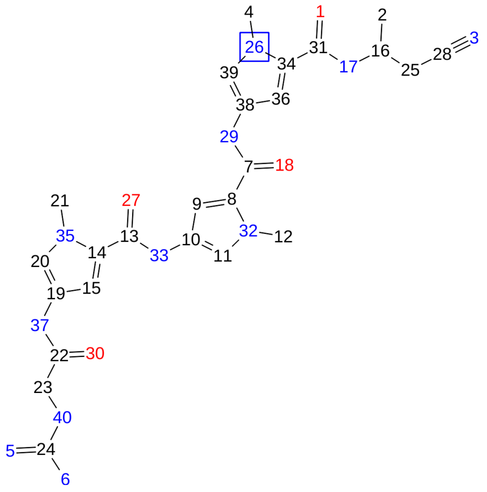

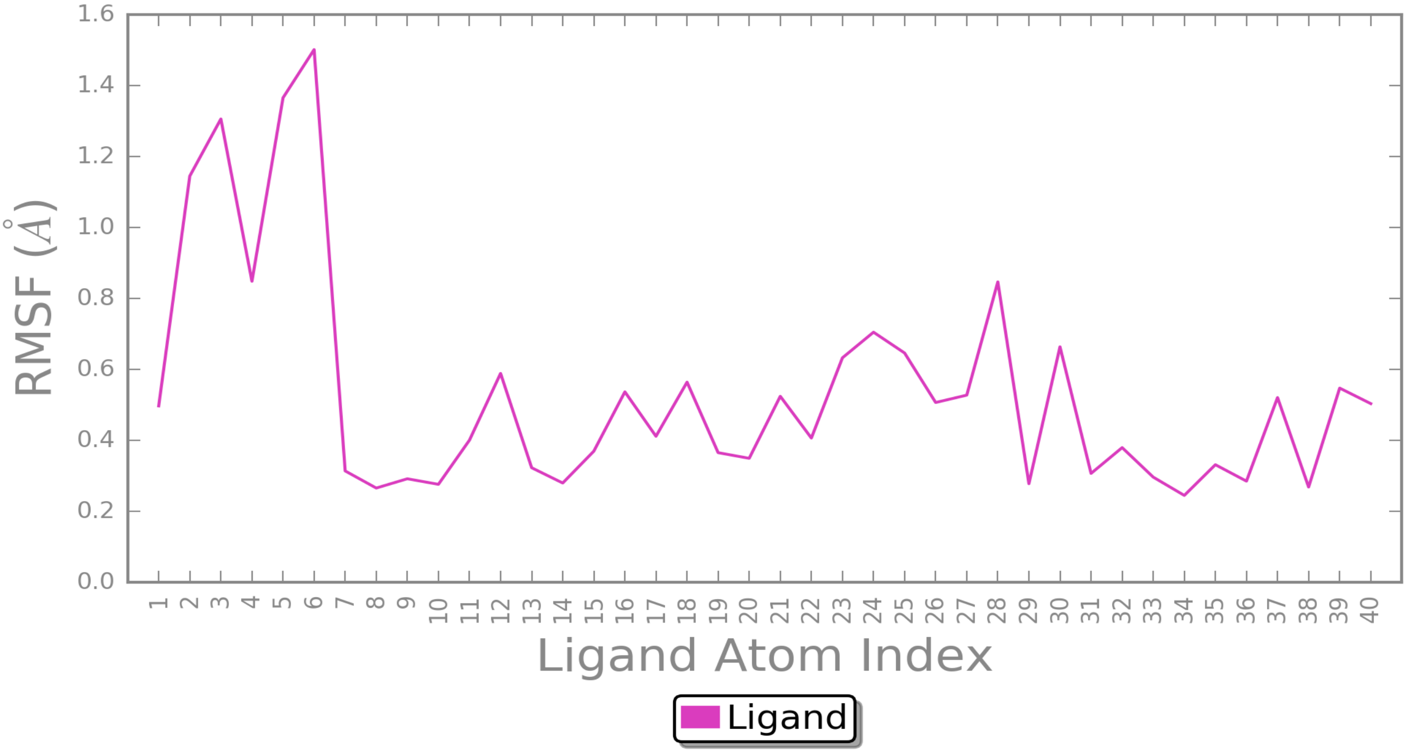

The Ligand Root Mean Square Fluctuation (L-RMSF) is useful for characterizing changes in the ligand atom positions. The RMSF for atom *i* is:

$$RMSF_i = \sqrt{\frac{1}{T}\sum_{t=1}^{T}(r'_i(t)) - r_i(t_{ref}))^2}$$

where T is the trajectory time over which the RMSF is calculated, t_{ref} is the reference time (usually for the first frame, and is regarded as the *zero* of time); r is the position of atom i in the reference at time t_{ref}, and r' is the position of atom i at time t after superposition on the reference frame.

Ligand RMSF shows the ligand's fluctuations broken down by atom, corresponding to the 2D structure in the top panel. The ligand RMSF may give you insights on how ligand fragments interact with the protein and their entropic role in the binding event. In the bottom panel, the 'Ligand' line shows fluctuations where the ligand in each frame is aligned on the ligand in the reference frame, and its fluctuations are measured for the ligand heavy atoms. These RMSF values reflect the internal atom fluctuations of the ligand.

Ligand Torsion Profile

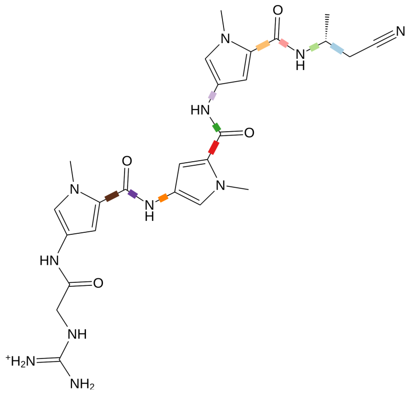

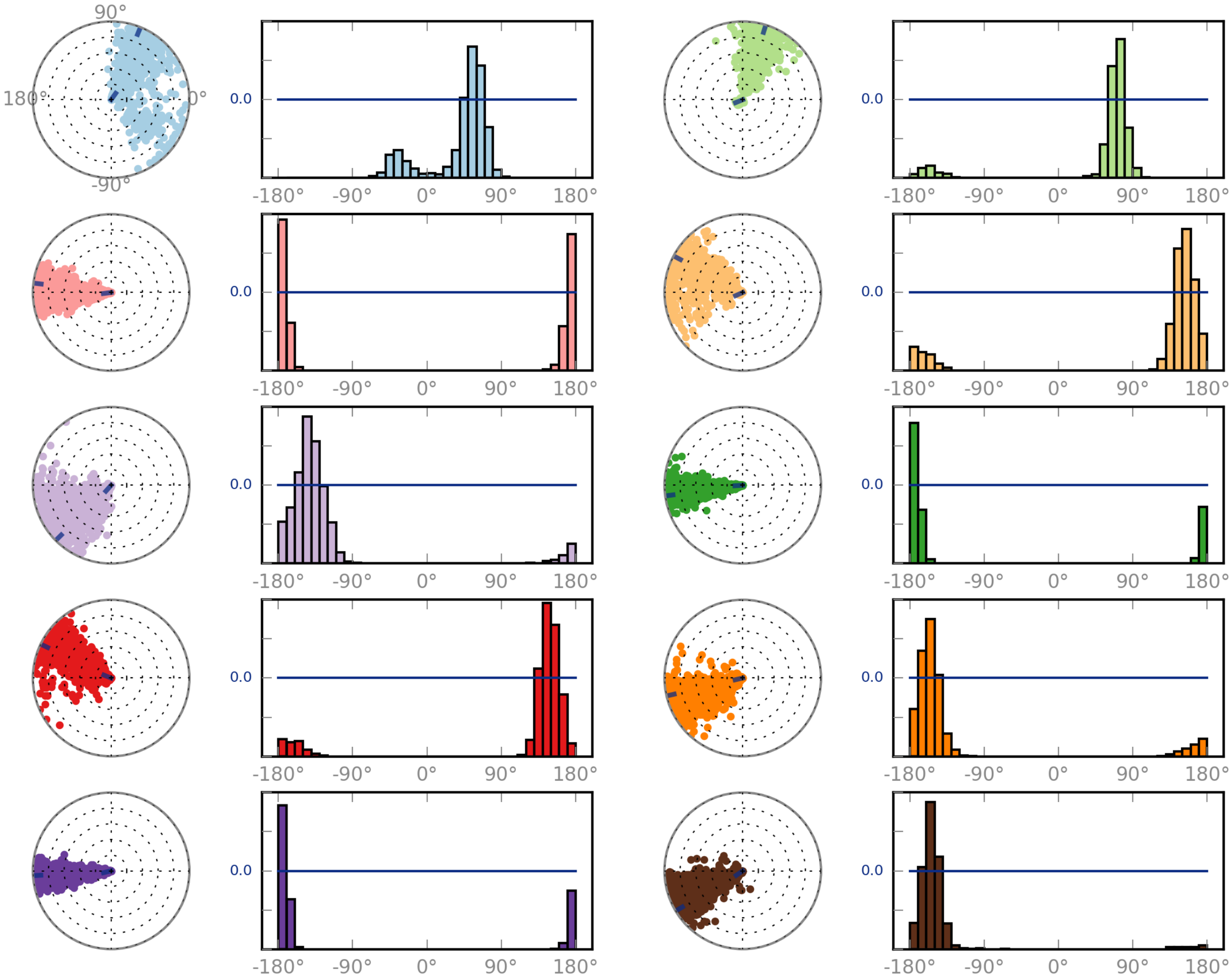

Ligand Torsion Profile (cont.)

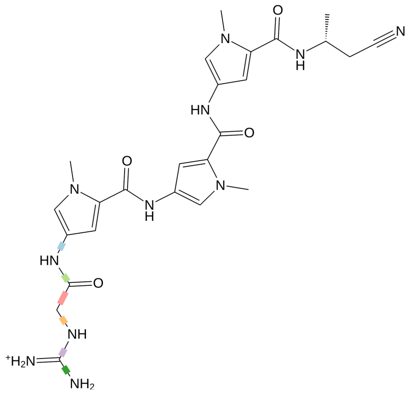

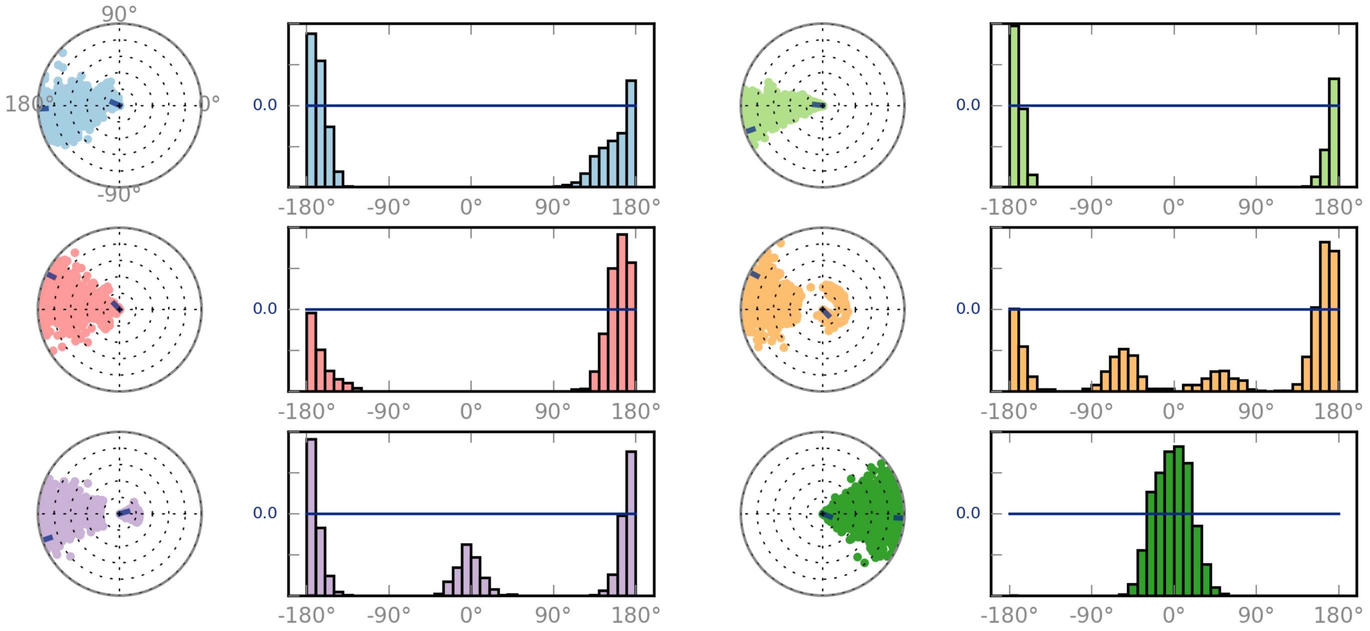

The ligand torsions plot summarizes the conformational evolution of every rotatable bond (RB) in the ligand throughout the simulation trajectory (0.00 through 5.00 nsec). The top panel shows the 2d schematic of a ligand with color-coded rotatable bonds. Each rotatable bond torsion is accompanied by a dial plot and bar plots of the same color.

Dial (or radial) plots describe the conformation of the torsion throughout the course of the simulation. The beginning of the simulation is in the center of the radial plot and the time evolution is plotted radially outwards.

The bar plots summarize the data on the dial plots, by showing the probability density of the torsion. If torsional potential information is available, the plot also shows the potential of the rotatable bond (by summing the potential of the related torsions). The values of the potential are on the left *Y*-axis of the chart, and are expressed in *kcal/mol*. Looking at the histogram and torsion potential relationships may give insights into the conformational strain the ligand undergoes to maintain a protein-bound conformation.

Ligand Properties

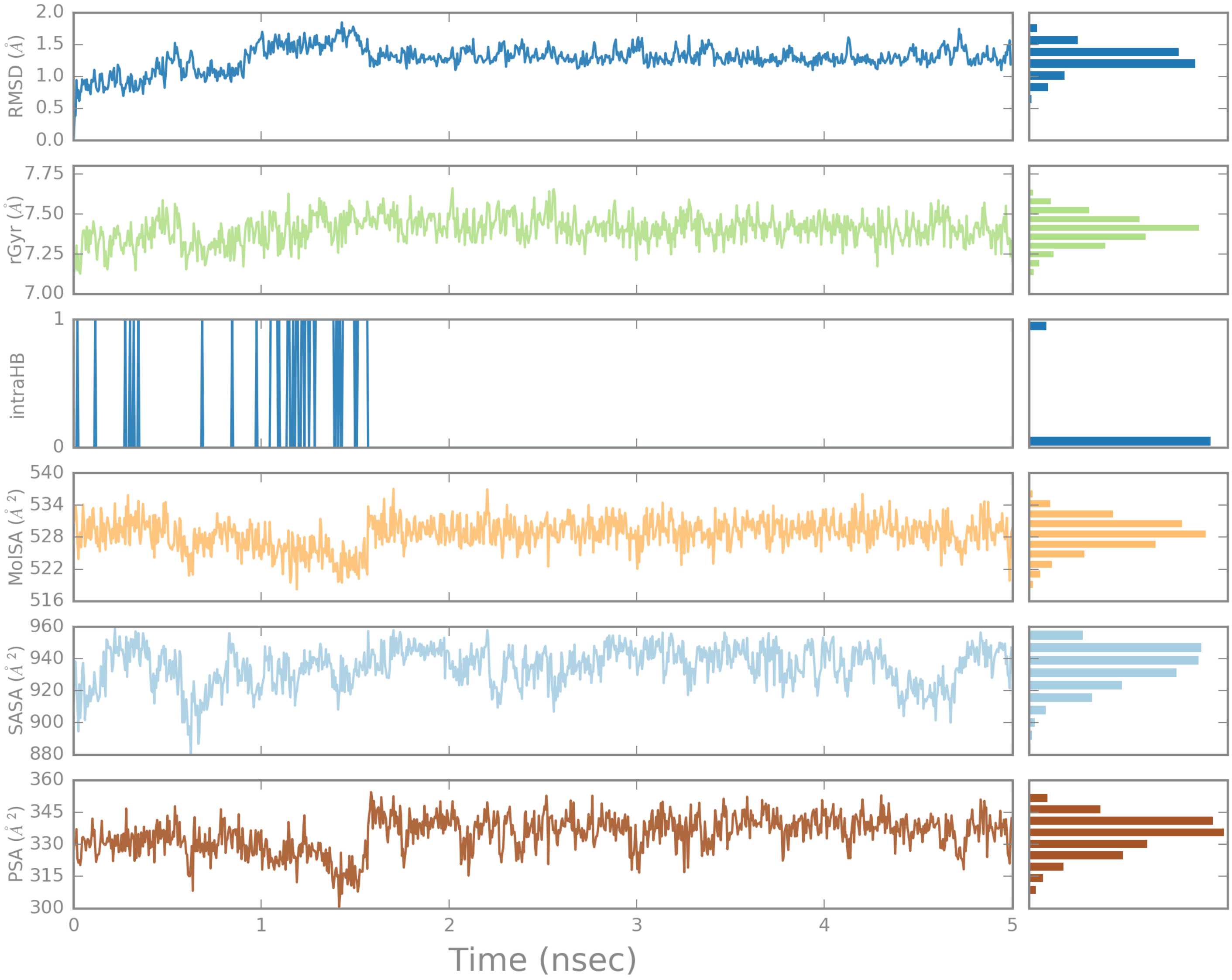

Ligand RMSD: Root mean square deviation of a ligand with respect to the reference conformation (typically the first frame is used as the reference and it is regarded as time *t*=0).

Radius of Gyration (rGyr): Measures the 'extendedness' of a ligand, and is equivalent to its principal moment of inertia.

Intramolecular Hydrogen Bonds (intraHB): Number of internal hydrogen bonds (HB) within a ligand molecule.

Molecular Surface Area (MolSA): Molecular surface calculation with 1.4 Å probe radius. This value is equivalent to a van der Waals surface area.

Solvent Accessible Surface Area (SASA): Surface area of a molecule accessible by a water molecule.

Polar Surface Area (PSA): Solvent accessible surface area in a molecule contributed only by oxygen and nitrogen atoms.
