# Supplementary figures and images for: Comparative in silico study of congocidine congeners as potential inhibitors of African swine fever virus
Source: PLoS One. 2019 Aug 28;14(8):e0221175. doi: 10.1371/journal.pone.0221175 (PMC6713398; doi:10.1371/journal.pone.0221175)

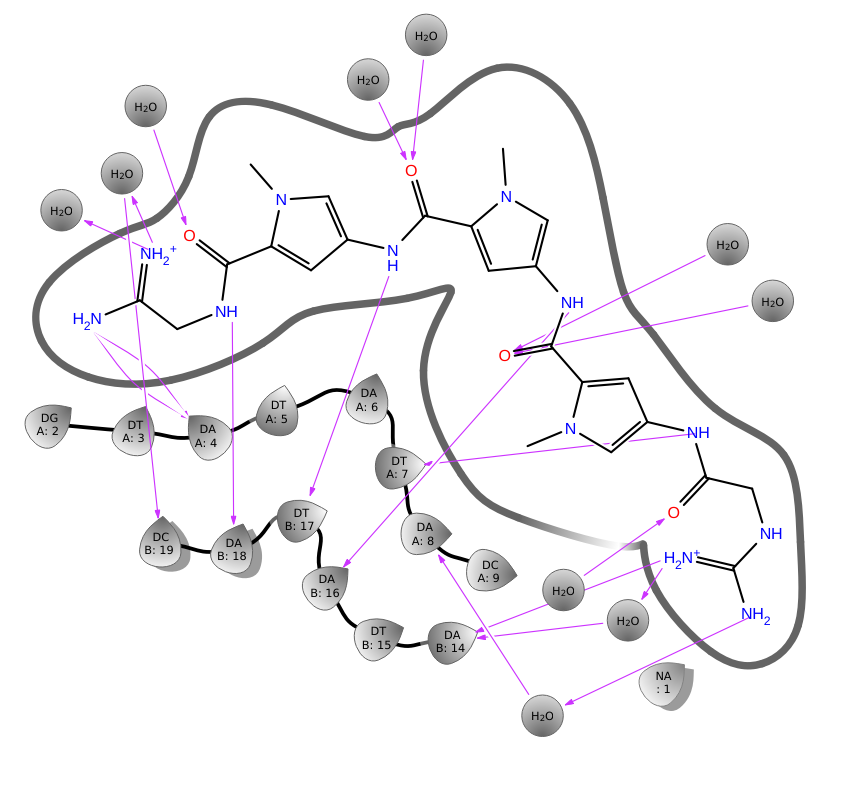

Supplement: S5 Data — (ZIP) [file pone.0221175.s005.zip › congocidine 2/con2f0.png]

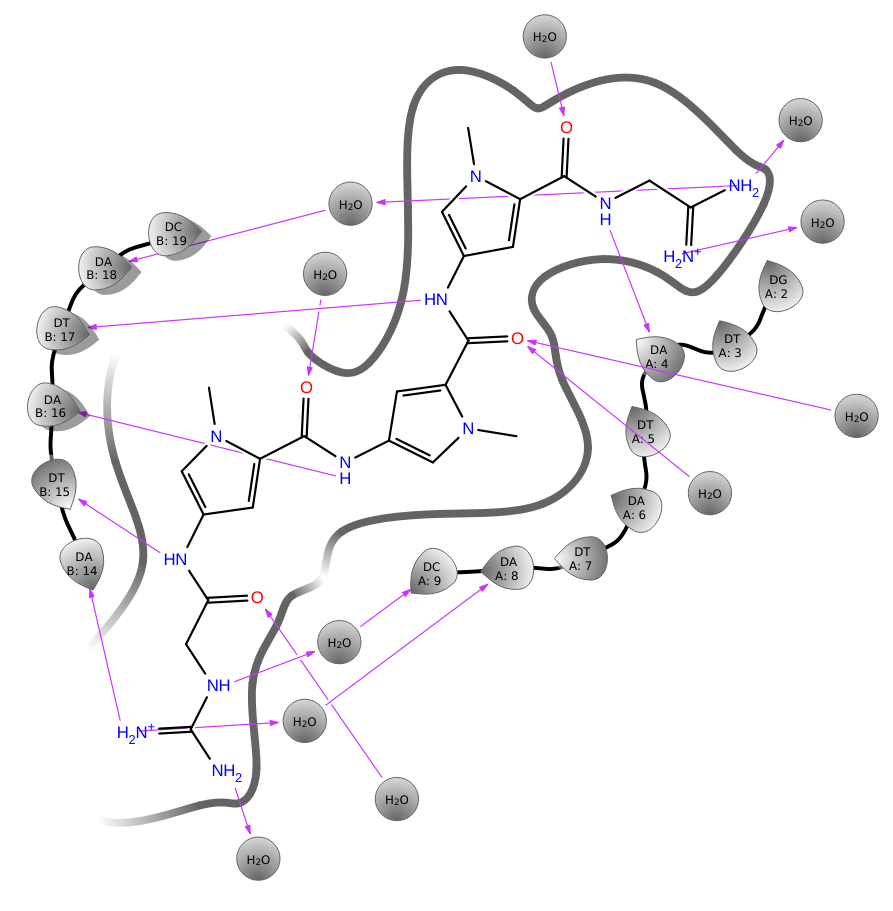

Supplement: S5 Data — (ZIP) [file pone.0221175.s005.zip › congocidine 2/con2f100.png]

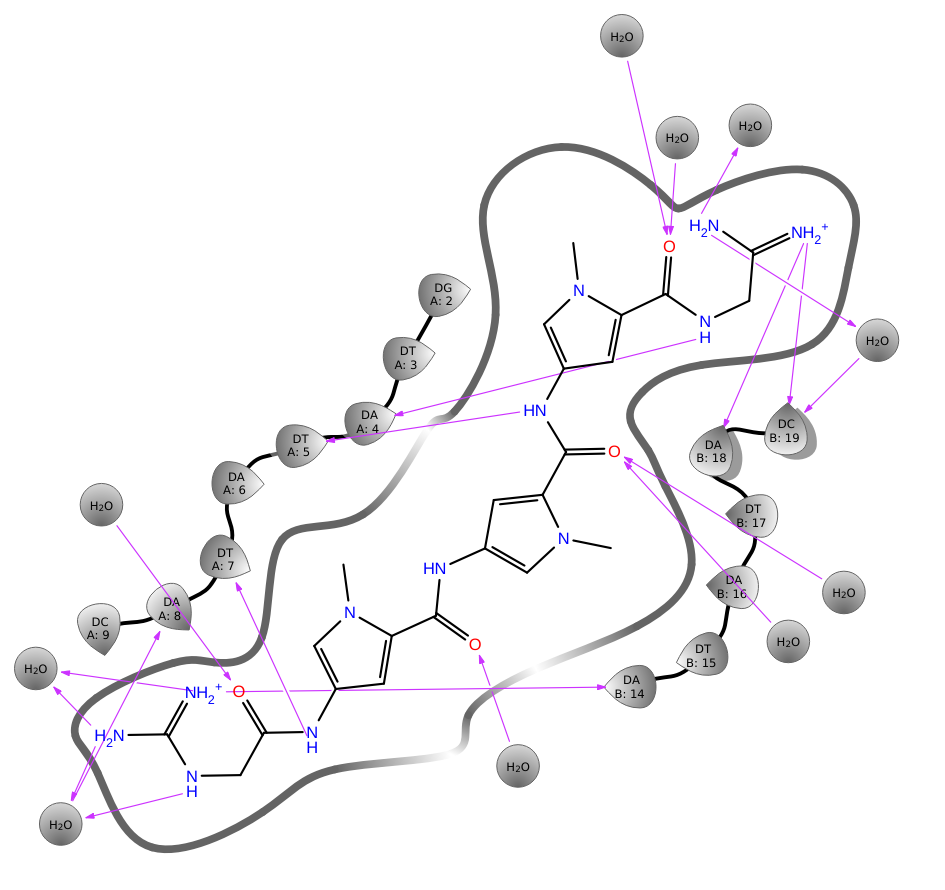

Supplement: S5 Data — (ZIP) [file pone.0221175.s005.zip › congocidine 2/con2f1000.png]

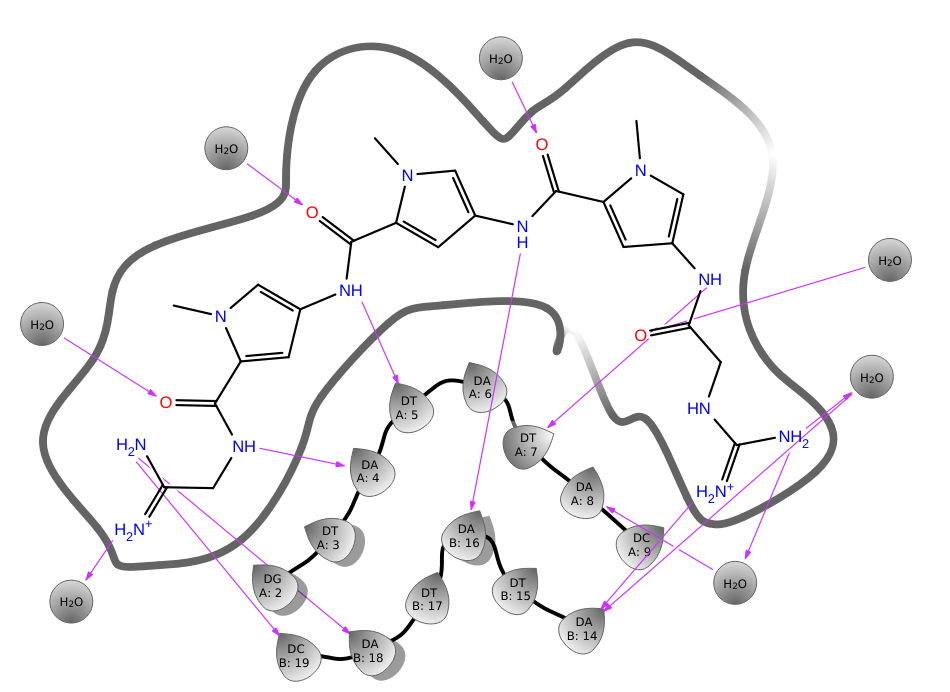

Supplement: S5 Data — (ZIP) [file pone.0221175.s005.zip › congocidine 2/con2f200.png]

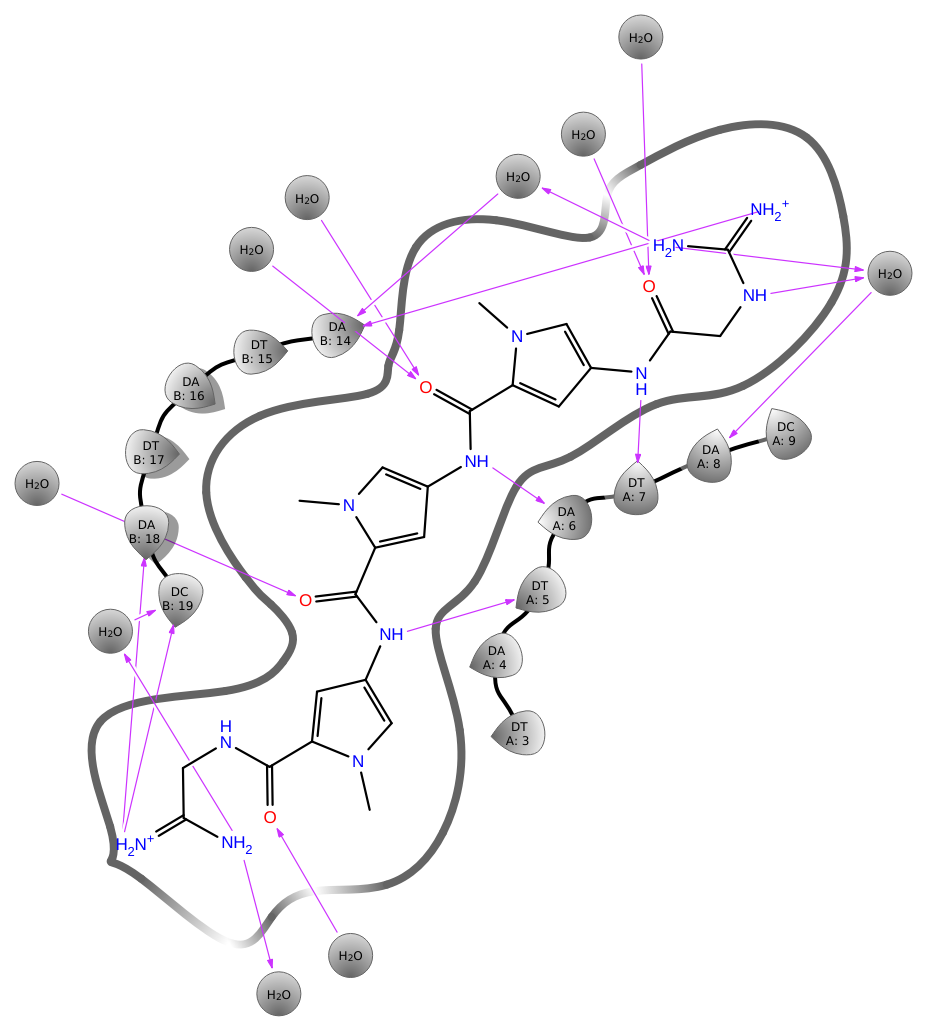

Supplement: S5 Data — (ZIP) [file pone.0221175.s005.zip › congocidine 2/con2f300.png]

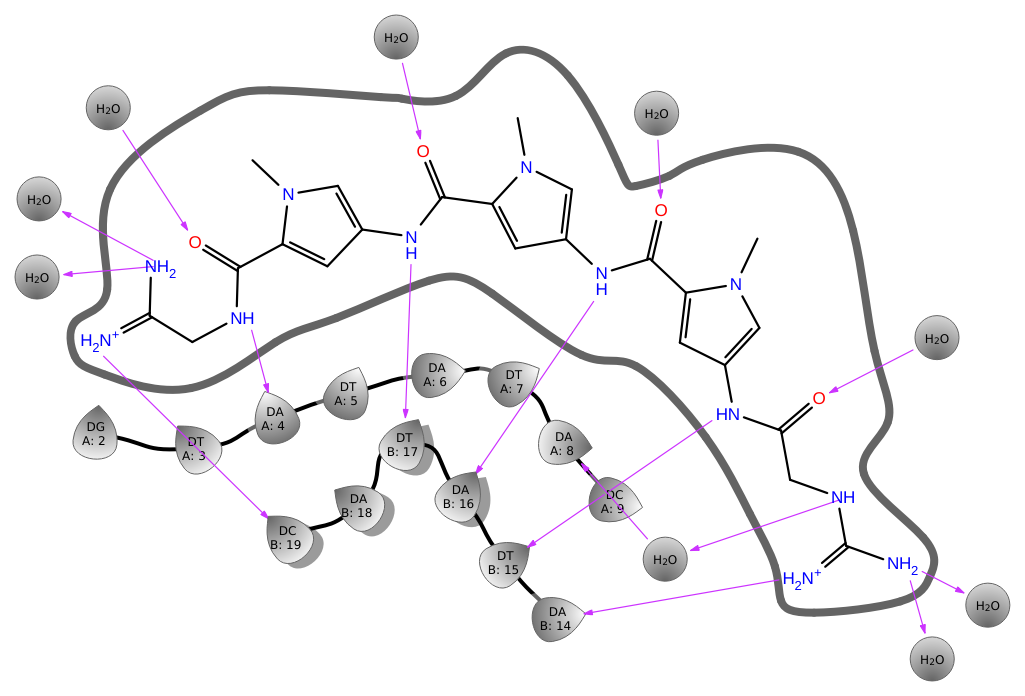

Supplement: S5 Data — (ZIP) [file pone.0221175.s005.zip › congocidine 2/con2f400.png]

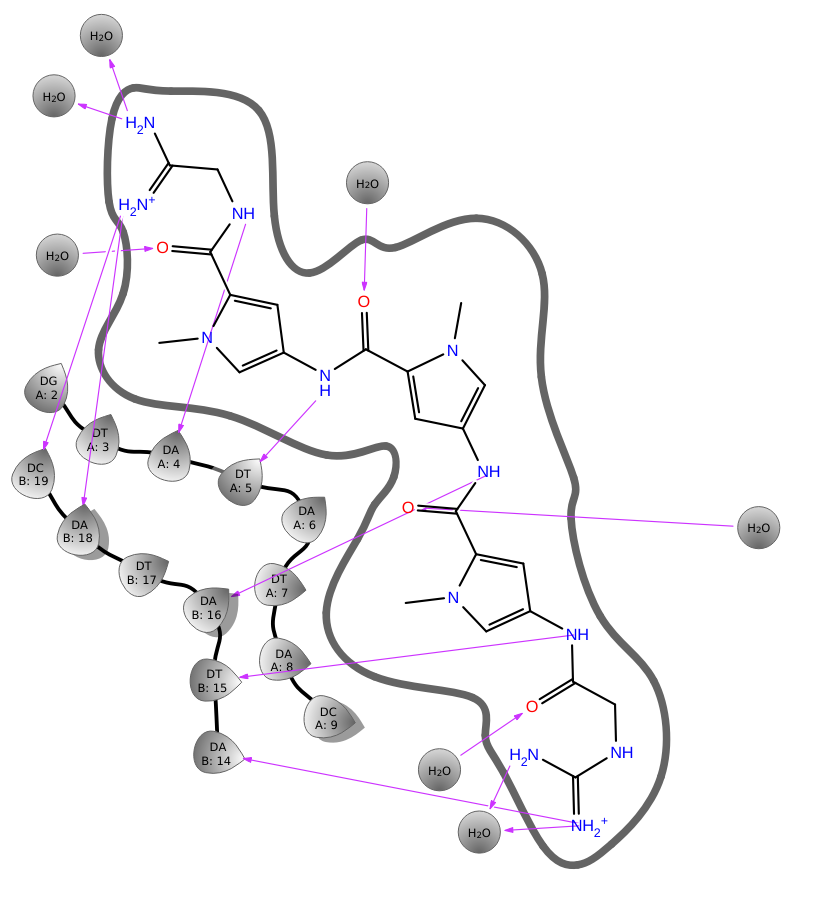

Supplement: S5 Data — (ZIP) [file pone.0221175.s005.zip › congocidine 2/con2f500.png]

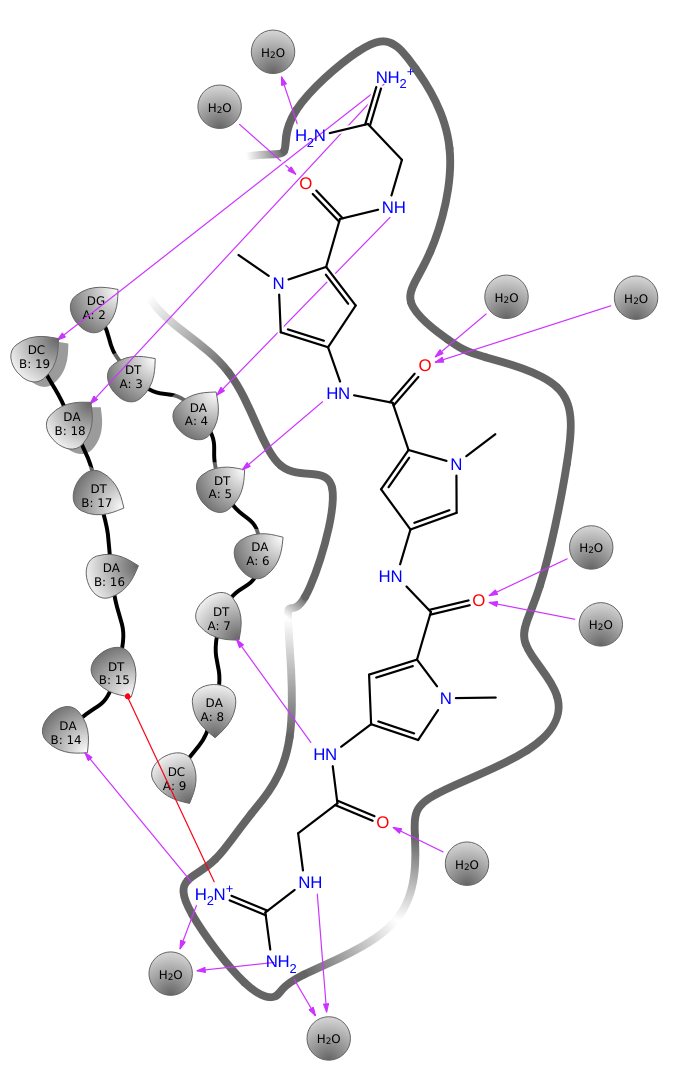

Supplement: S5 Data — (ZIP) [file pone.0221175.s005.zip › congocidine 2/con2f600.png]

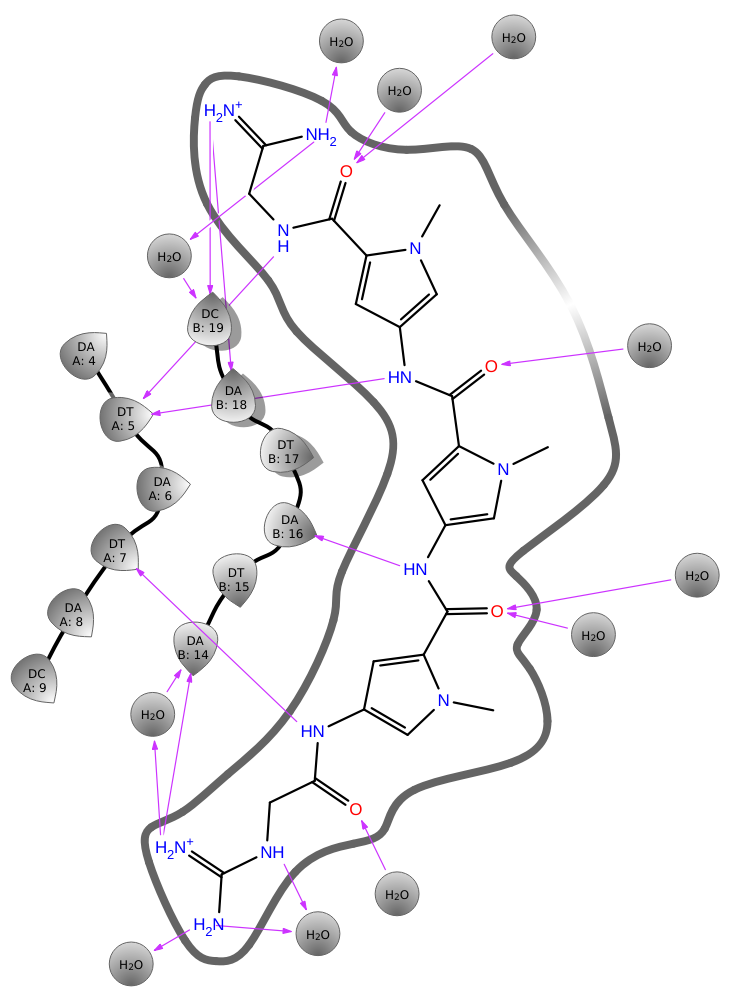

Supplement: S5 Data — (ZIP) [file pone.0221175.s005.zip › congocidine 2/con2f700.png]

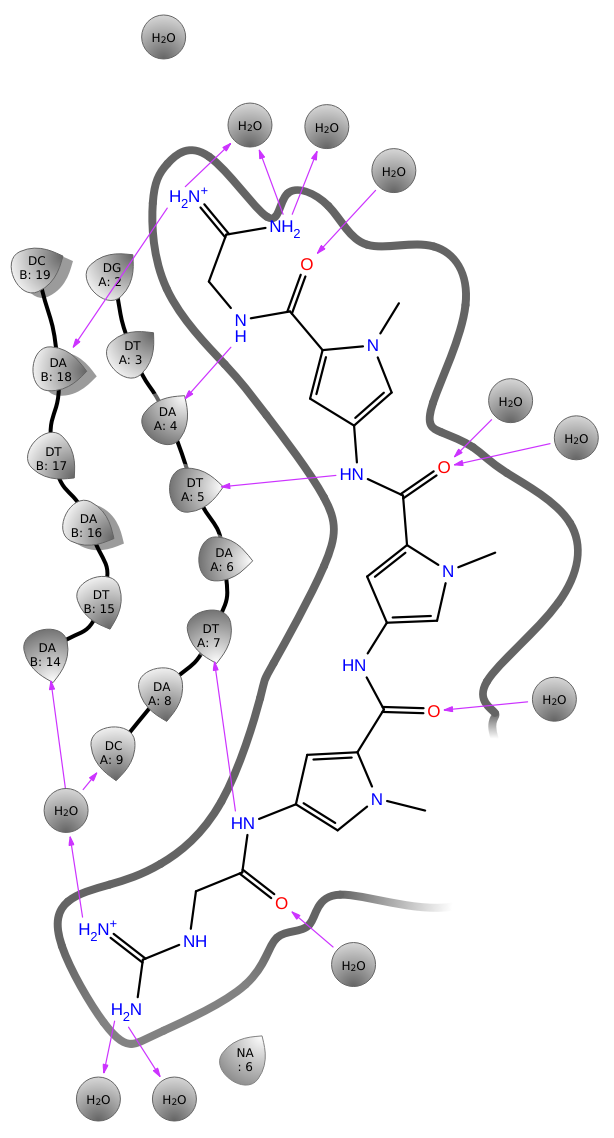

Supplement: S5 Data — (ZIP) [file pone.0221175.s005.zip › congocidine 2/con2f800.png]

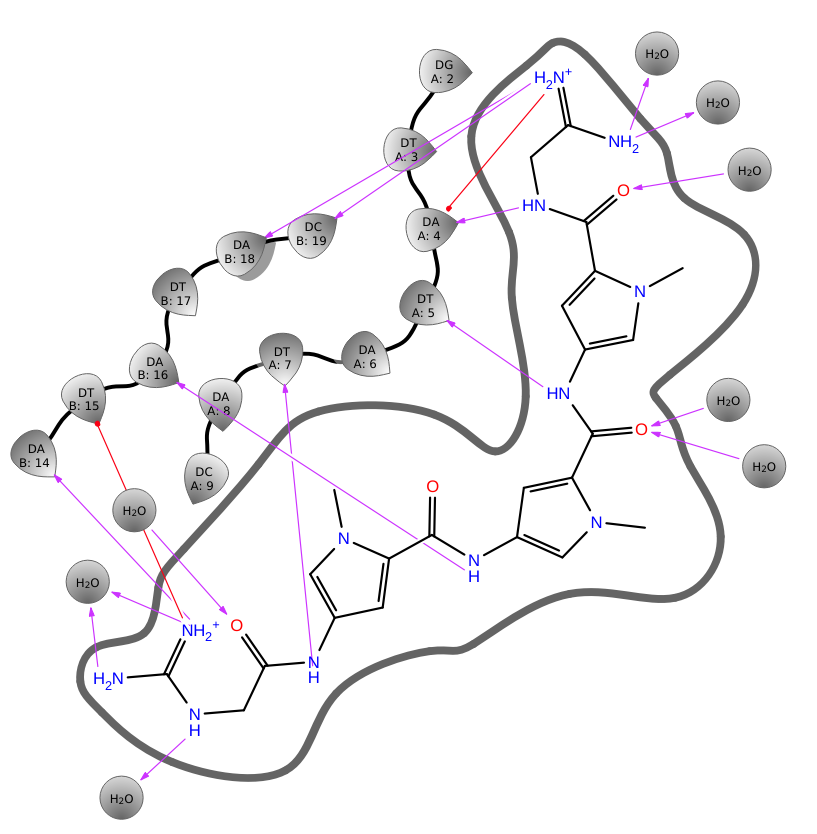

Supplement: S5 Data — (ZIP) [file pone.0221175.s005.zip › congocidine 2/con2f900.png]

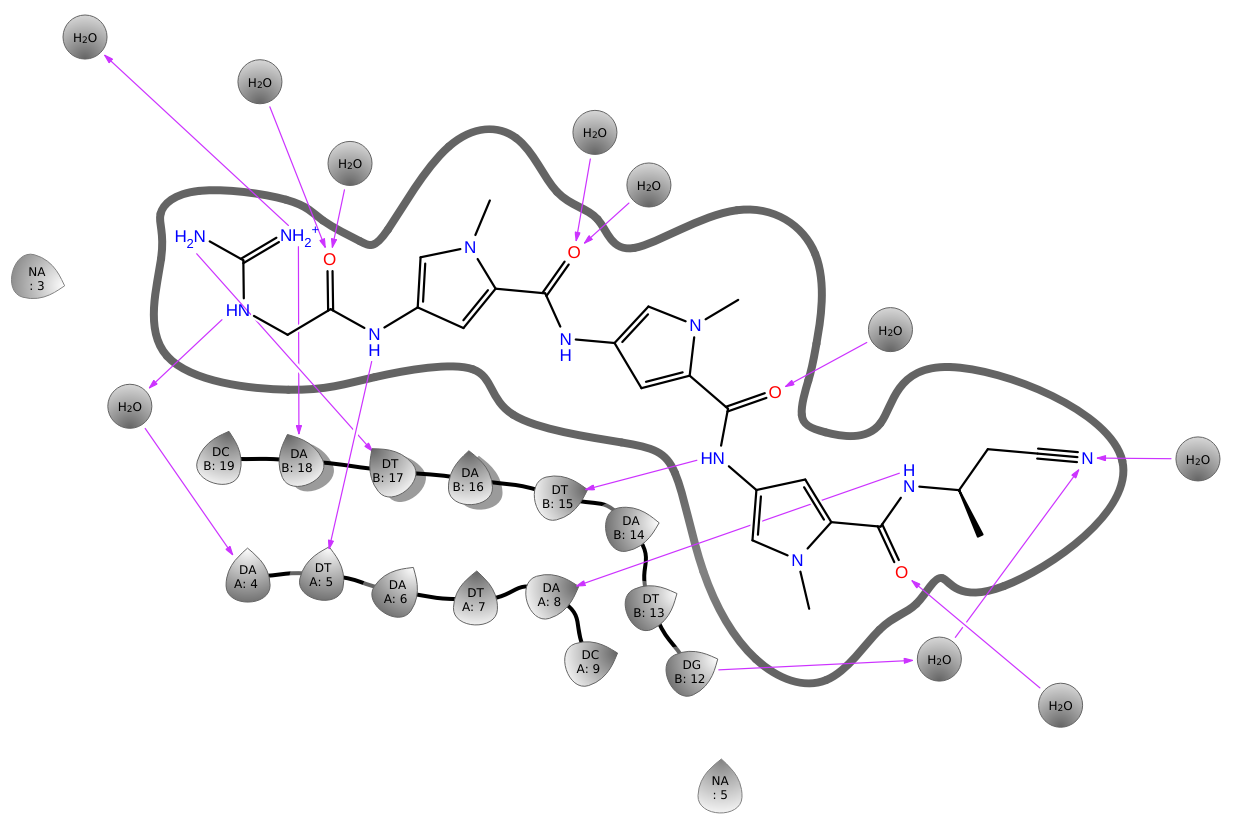

Supplement: S5 Data — (ZIP) [file pone.0221175.s005.zip › congocidine 3/congo3f0.png]

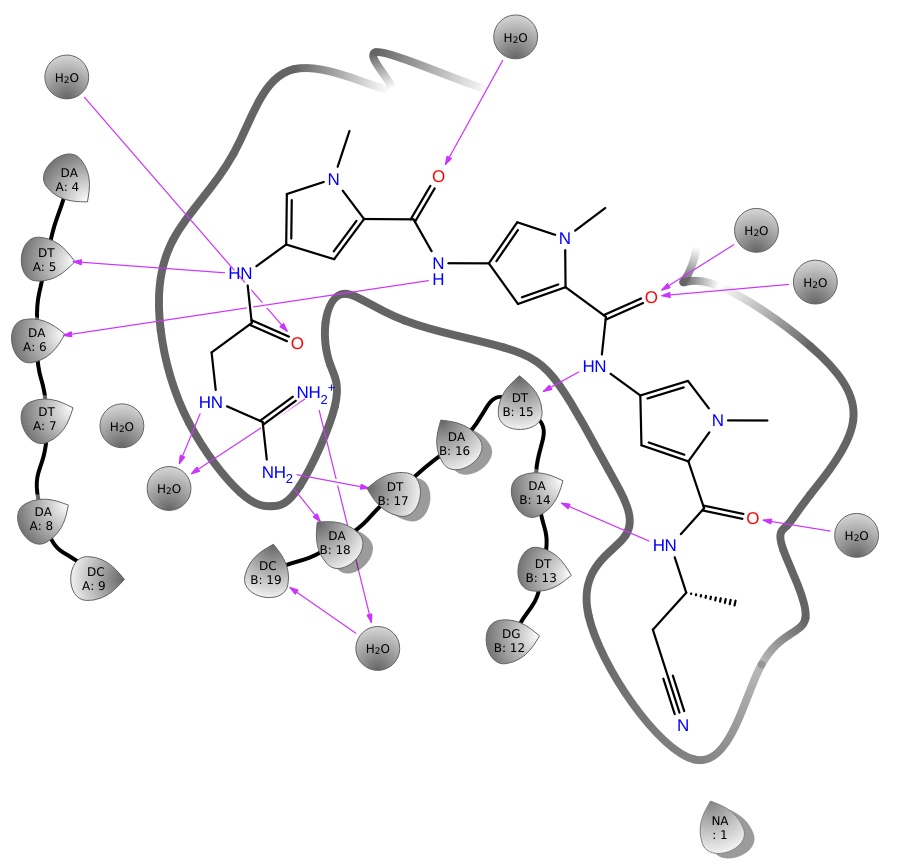

Supplement: S5 Data — (ZIP) [file pone.0221175.s005.zip › congocidine 3/congo3f100.png]

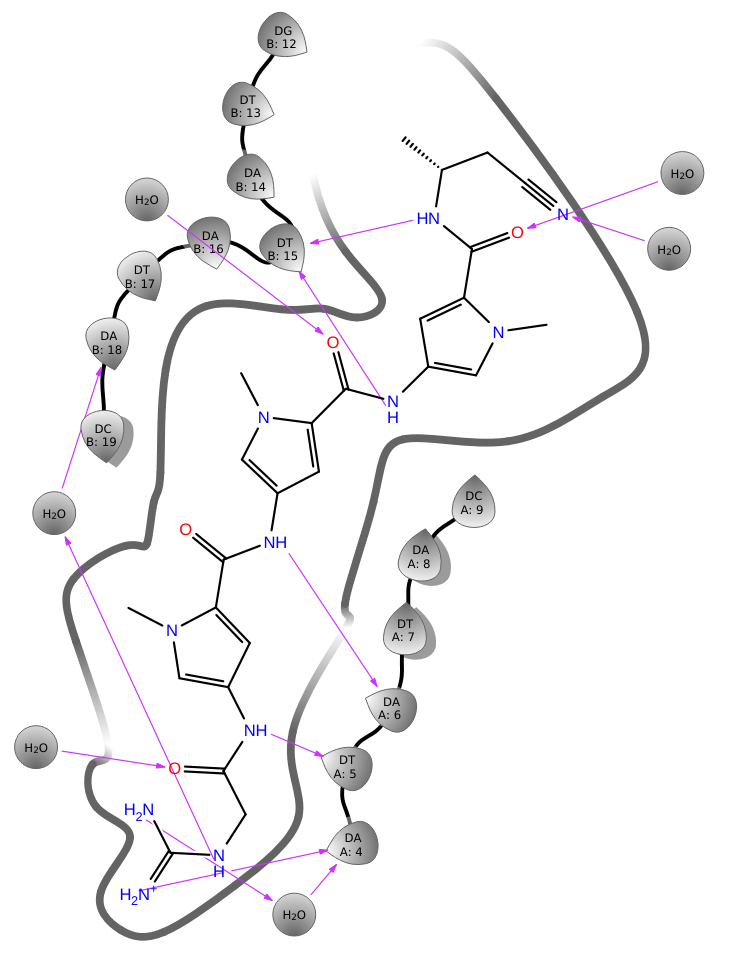

Supplement: S5 Data — (ZIP) [file pone.0221175.s005.zip › congocidine 3/congo3f1000.png]

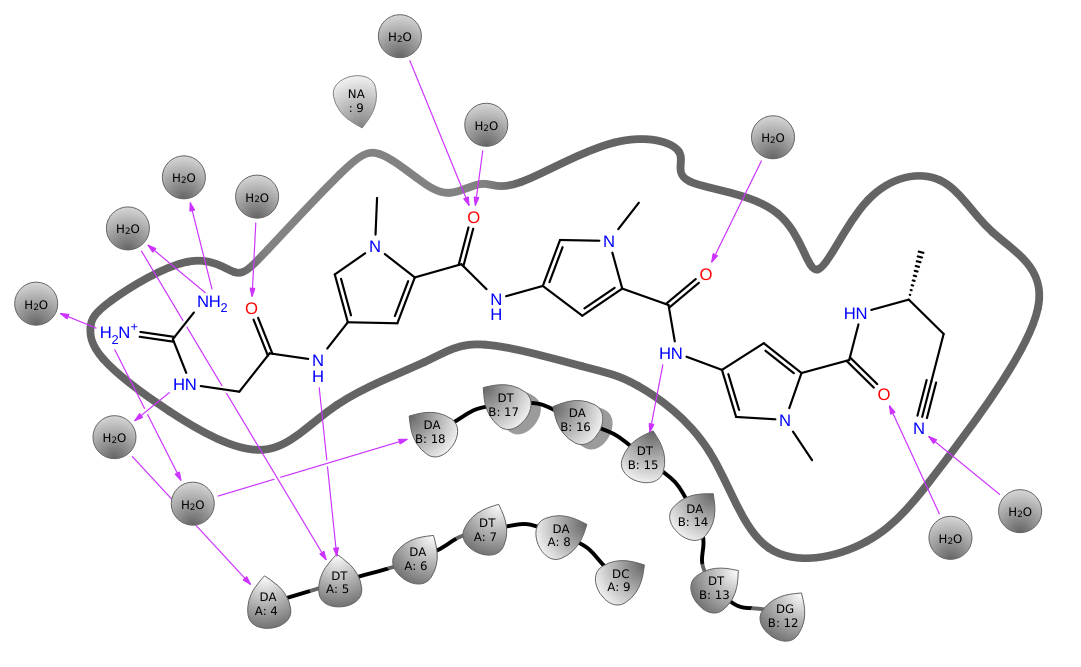

Supplement: S5 Data — (ZIP) [file pone.0221175.s005.zip › congocidine 3/congo3f200.png]

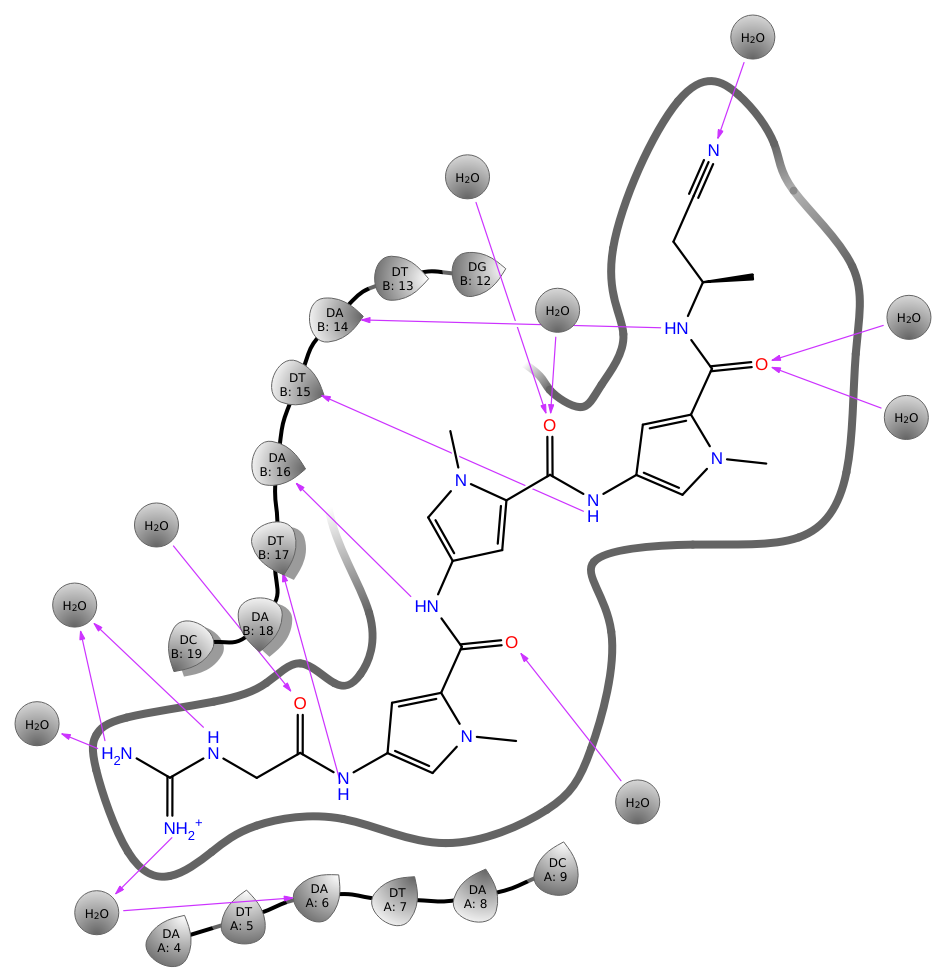

Supplement: S5 Data — (ZIP) [file pone.0221175.s005.zip › congocidine 3/congo3f300.png]

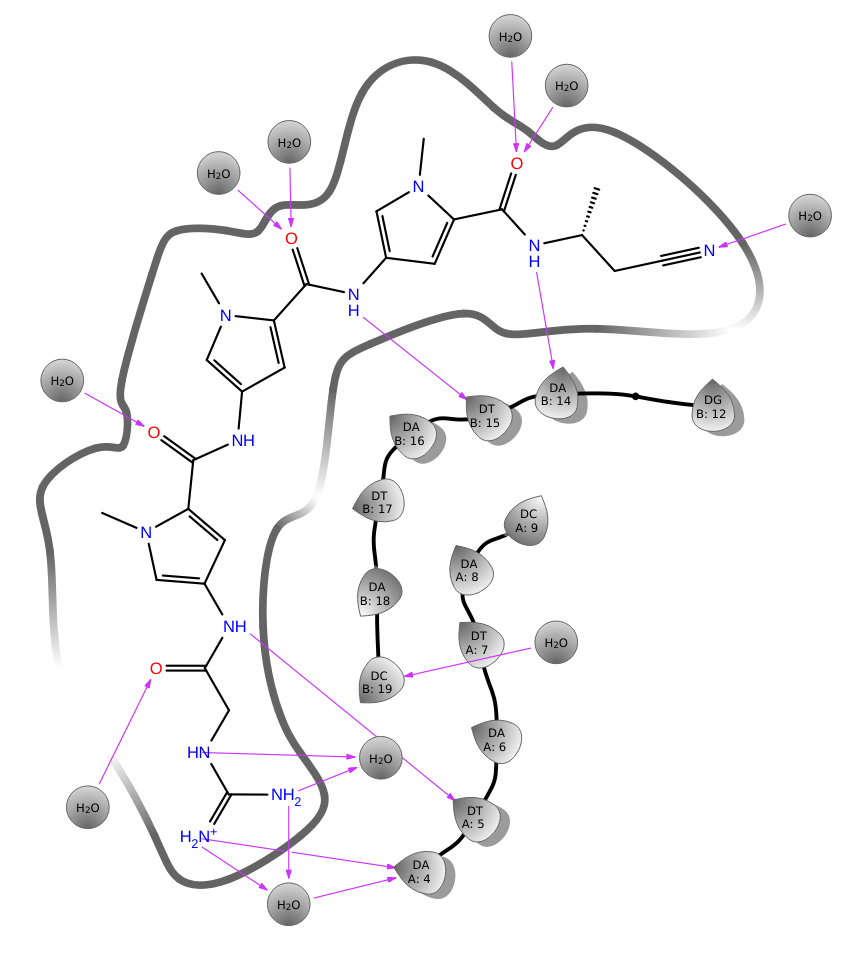

Supplement: S5 Data — (ZIP) [file pone.0221175.s005.zip › congocidine 3/congo3f400.png]

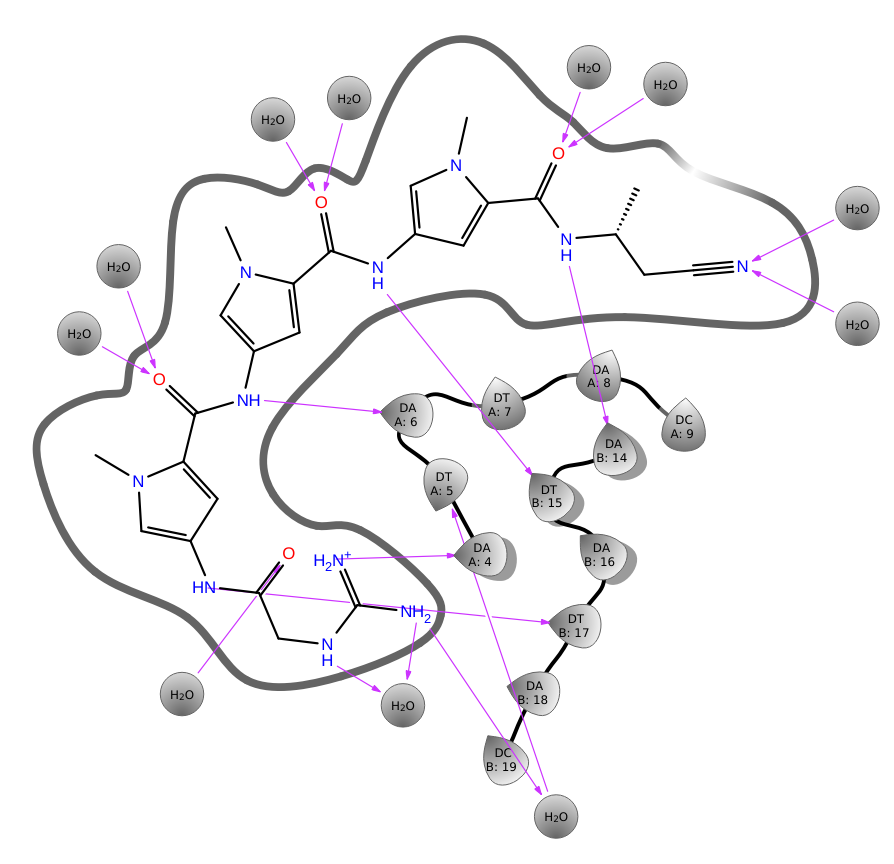

Supplement: S5 Data — (ZIP) [file pone.0221175.s005.zip › congocidine 3/congo3f500.png]

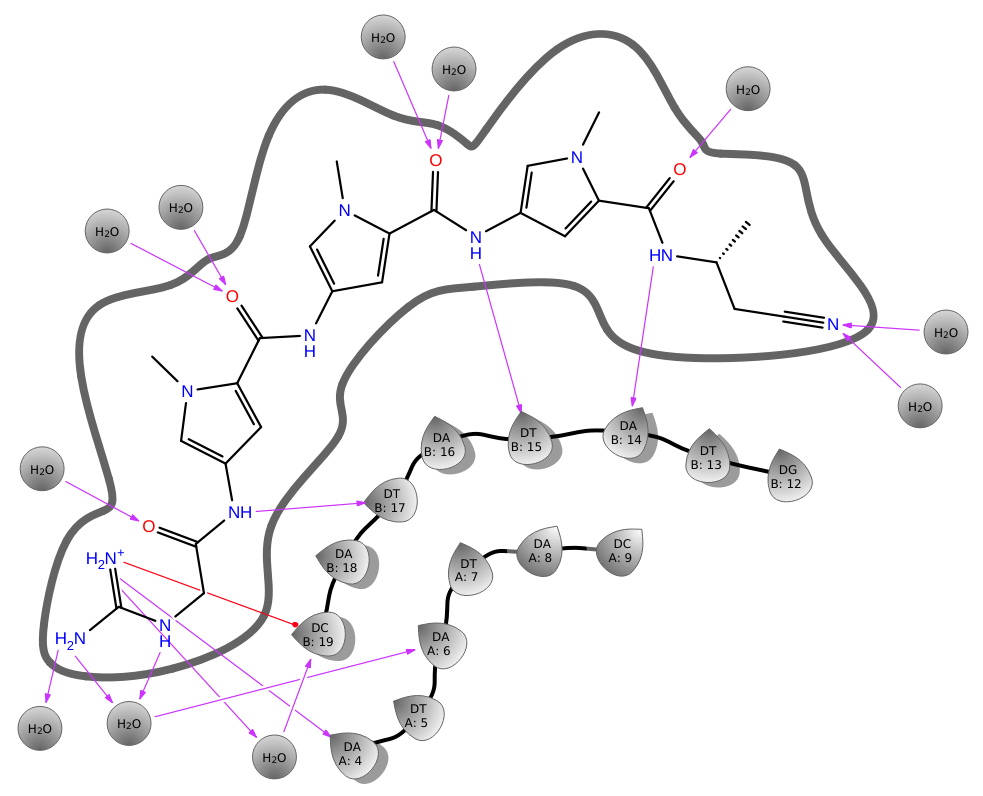

Supplement: S5 Data — (ZIP) [file pone.0221175.s005.zip › congocidine 3/congo3f600.png]

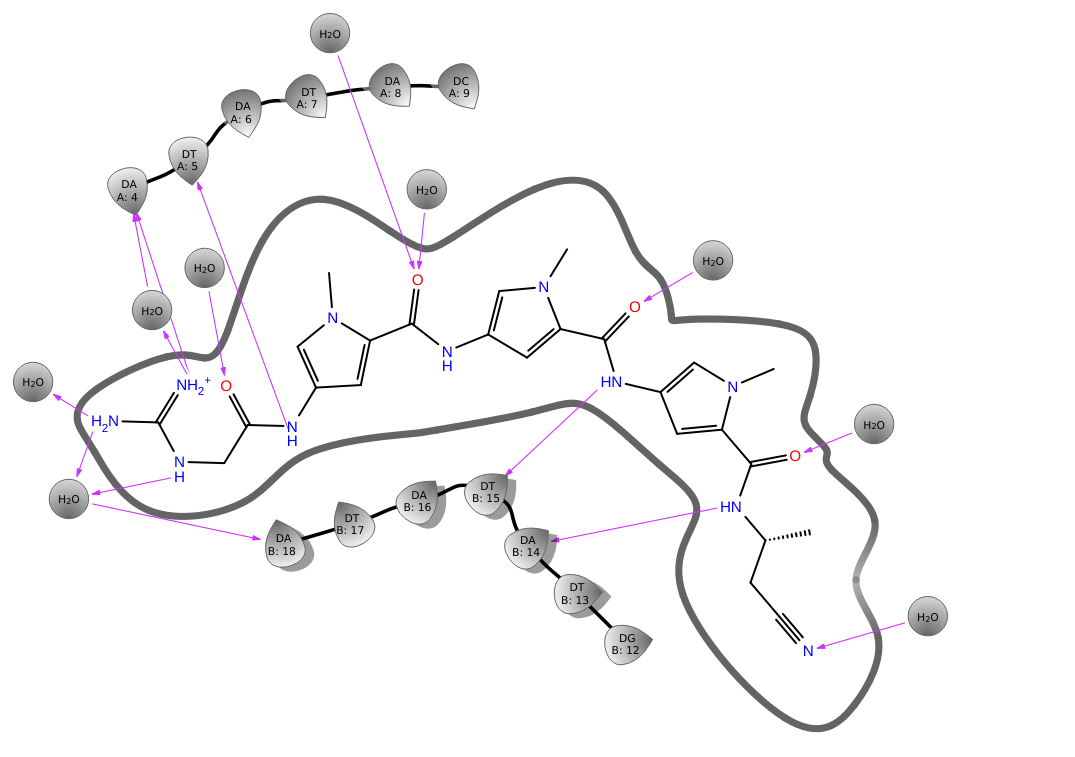

Supplement: S5 Data — (ZIP) [file pone.0221175.s005.zip › congocidine 3/congo3f700.png]

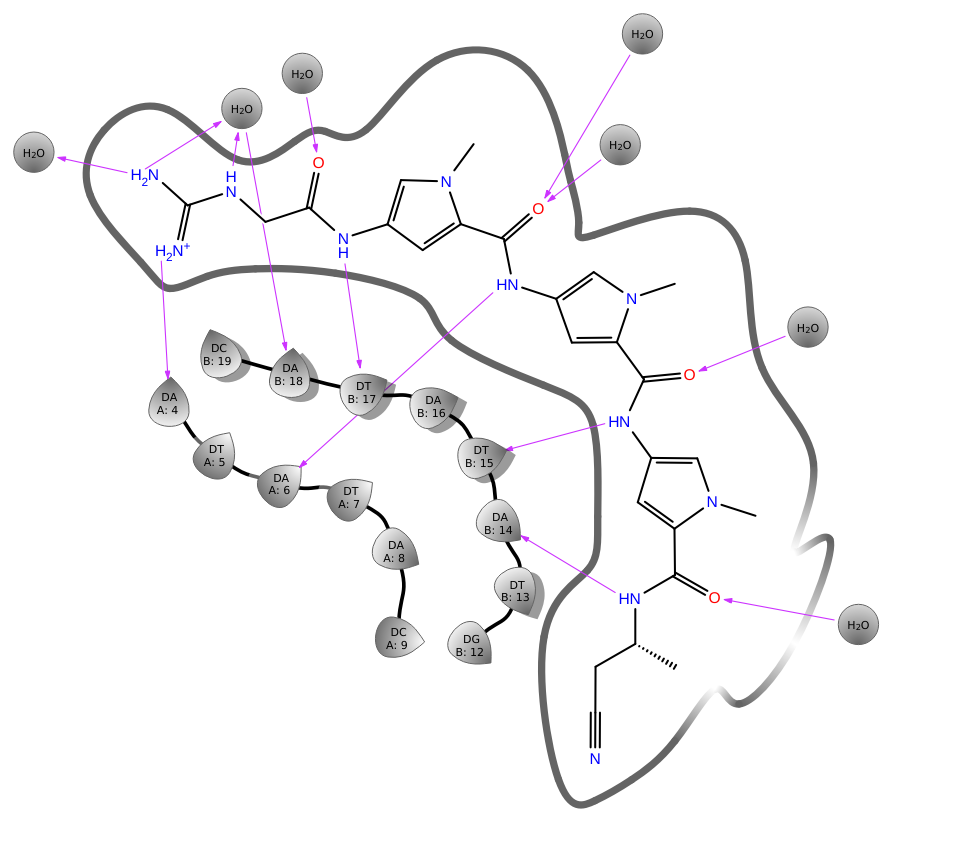

Supplement: S5 Data — (ZIP) [file pone.0221175.s005.zip › congocidine 3/congo3f800.png]

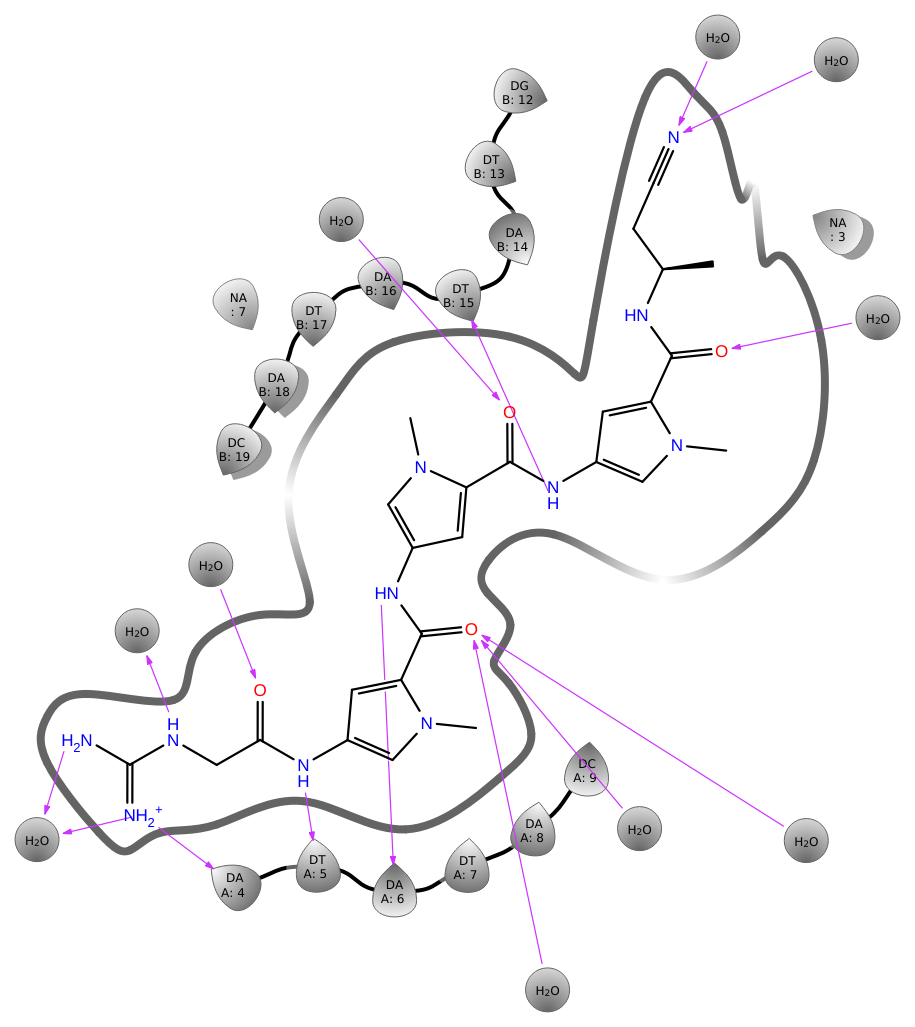

Supplement: S5 Data — (ZIP) [file pone.0221175.s005.zip › congocidine 3/congo3f900.png]

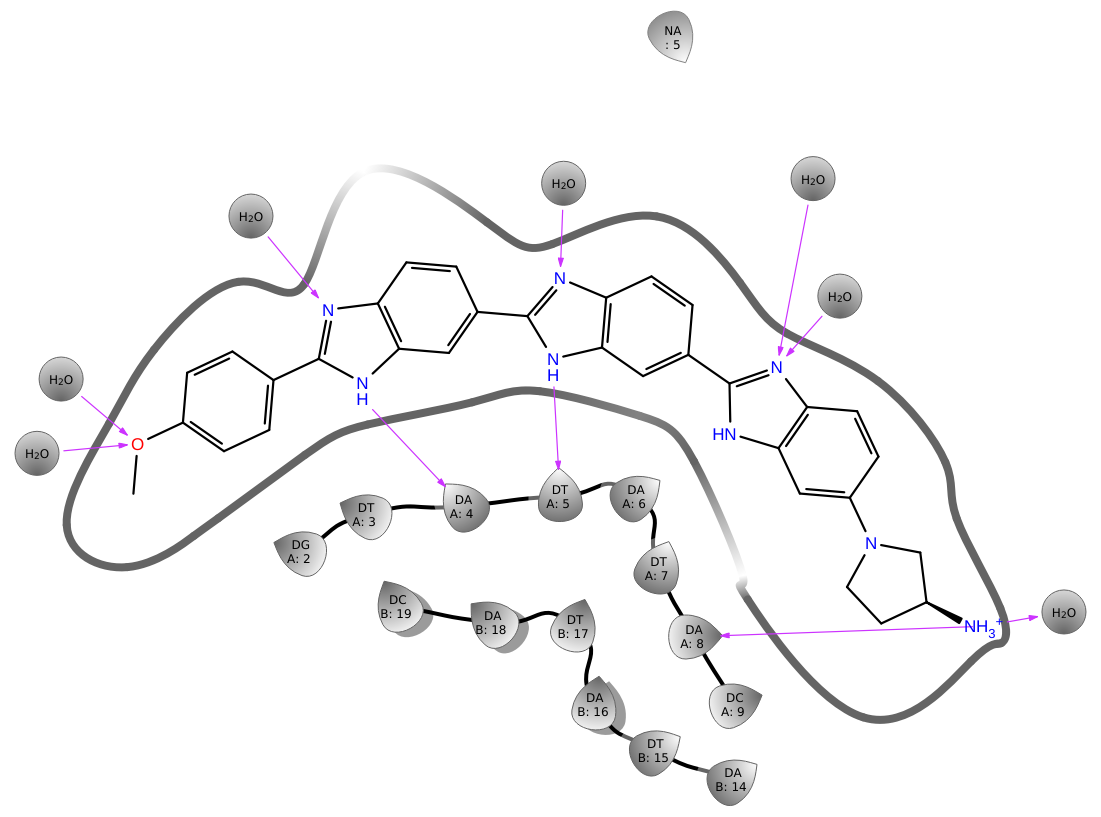

Supplement: S5 Data — (ZIP) [file pone.0221175.s005.zip › trisbenzimidazole/tribz000.png]

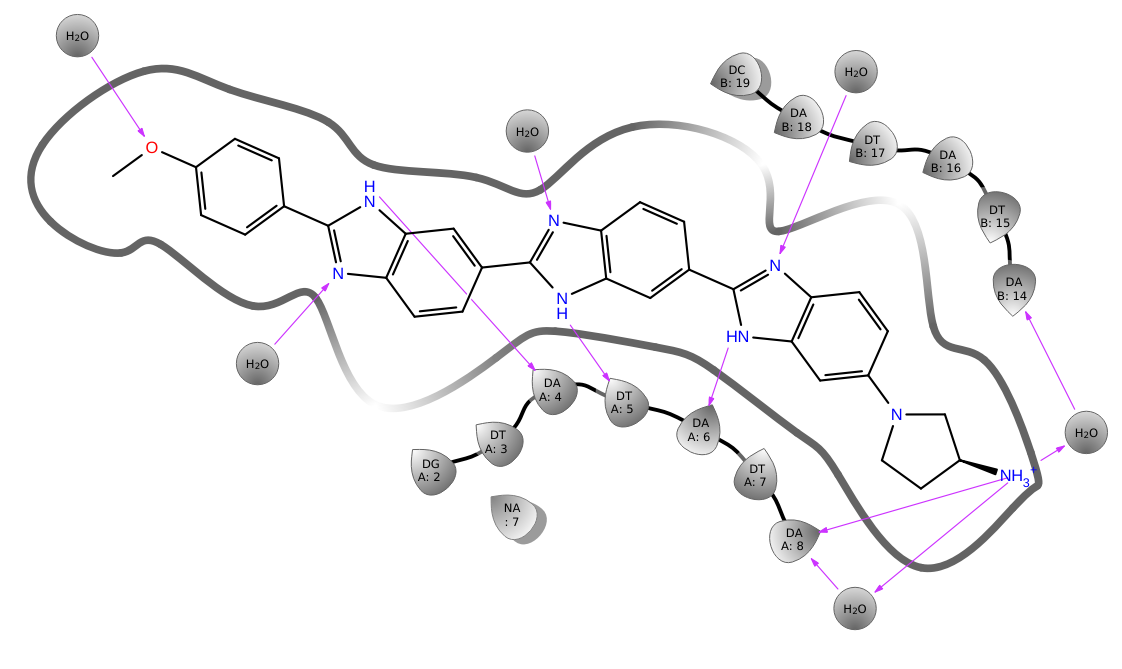

Supplement: S5 Data — (ZIP) [file pone.0221175.s005.zip › trisbenzimidazole/tribz100.png]

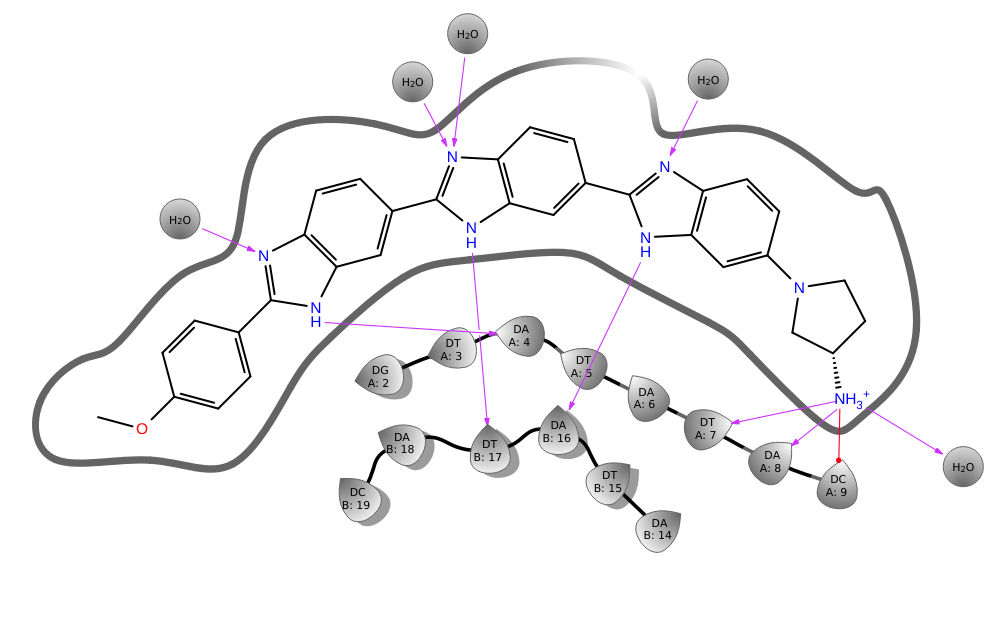

Supplement: S5 Data — (ZIP) [file pone.0221175.s005.zip › trisbenzimidazole/tribz1000.png]

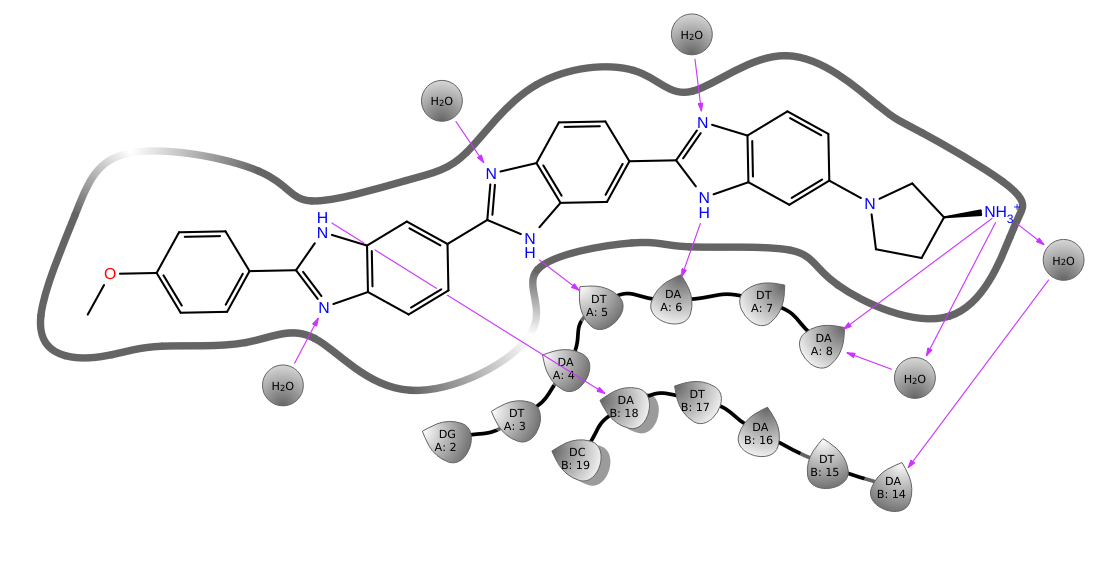

Supplement: S5 Data — (ZIP) [file pone.0221175.s005.zip › trisbenzimidazole/tribz200.png]

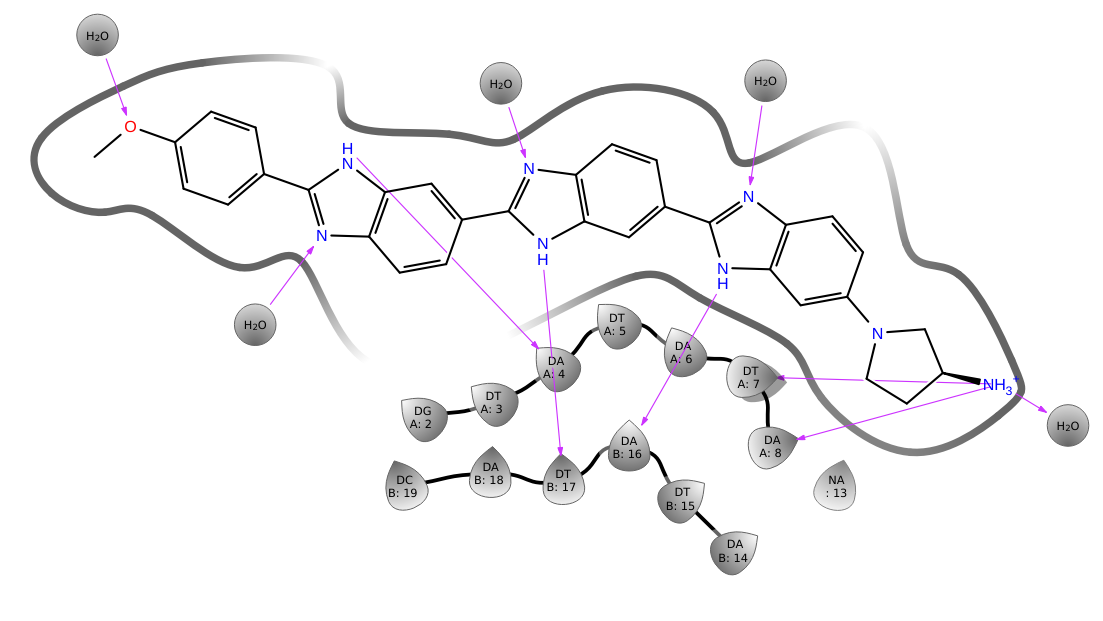

Supplement: S5 Data — (ZIP) [file pone.0221175.s005.zip › trisbenzimidazole/tribz300.png]

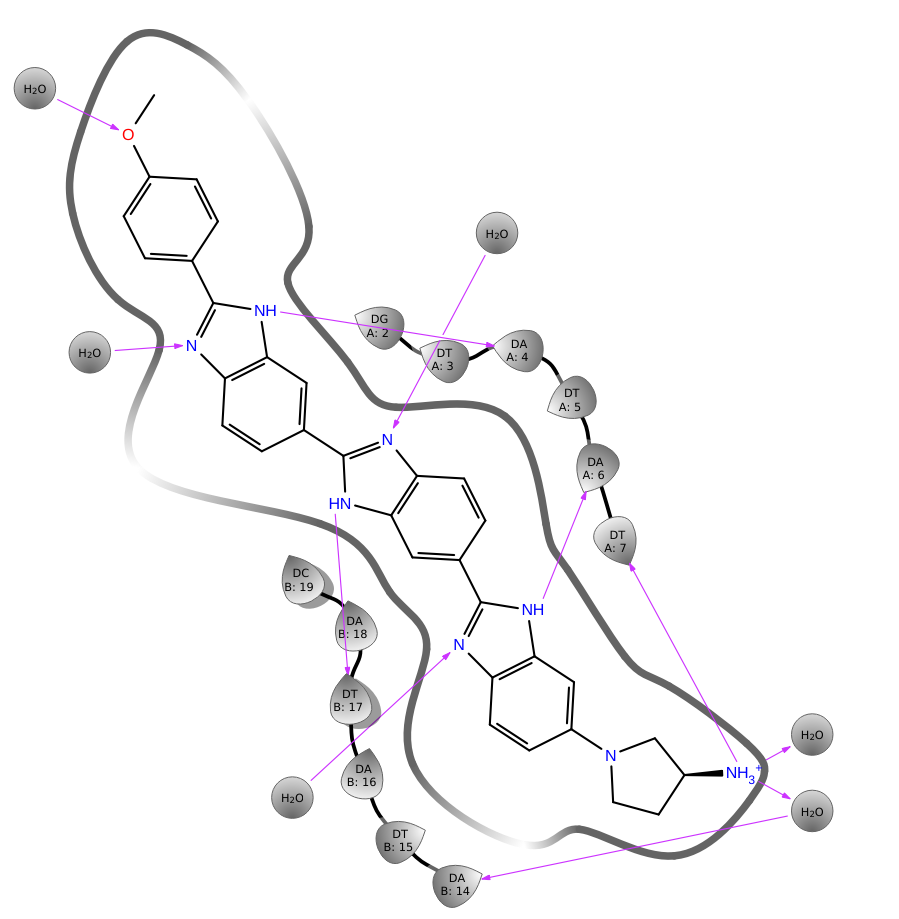

Supplement: S5 Data — (ZIP) [file pone.0221175.s005.zip › trisbenzimidazole/tribz400.png]

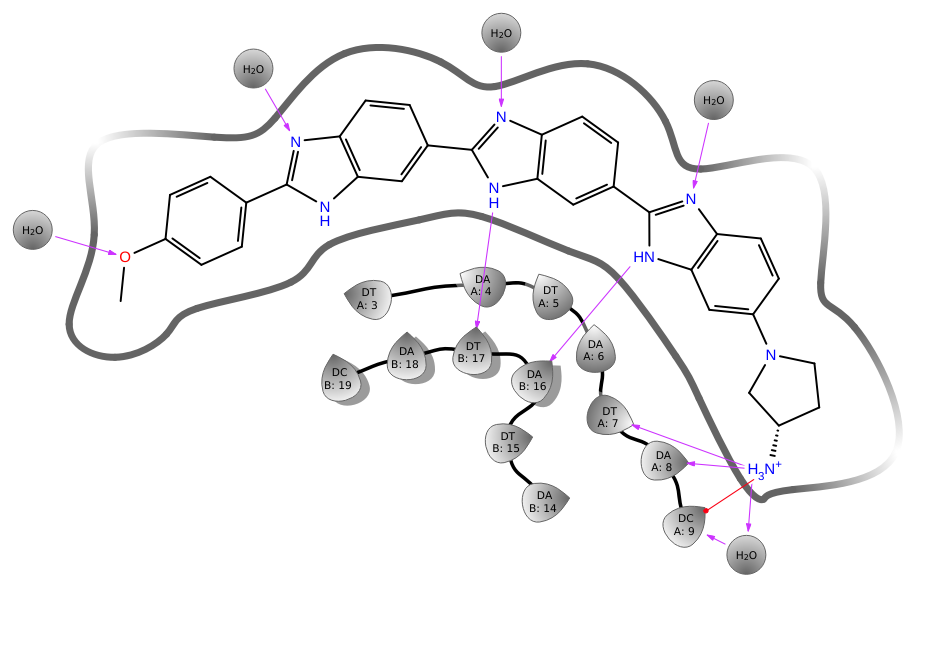

Supplement: S5 Data — (ZIP) [file pone.0221175.s005.zip › trisbenzimidazole/tribz500.png]

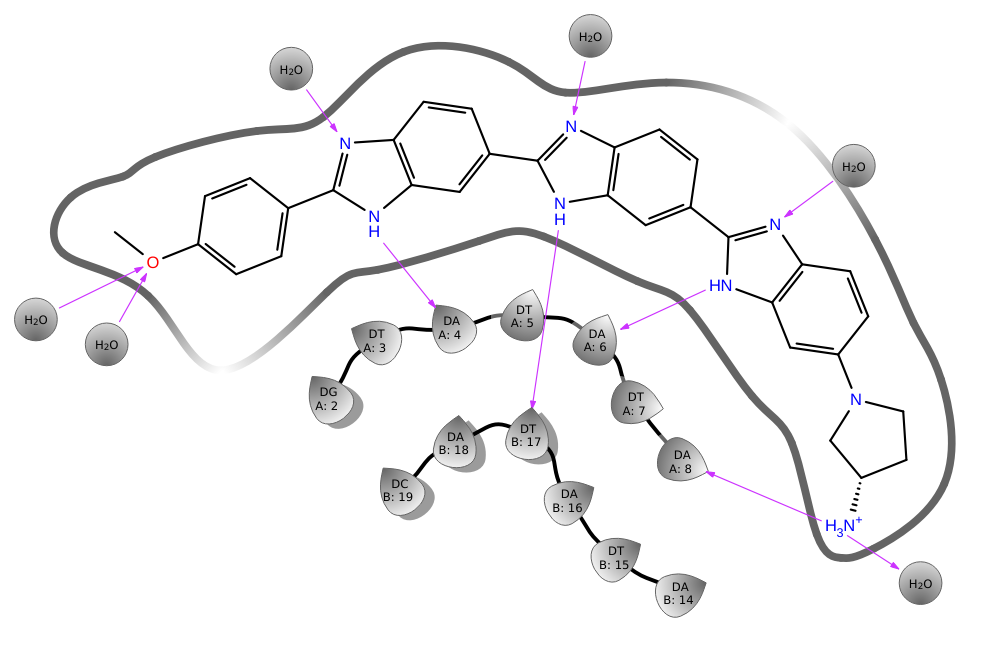

Supplement: S5 Data — (ZIP) [file pone.0221175.s005.zip › trisbenzimidazole/tribz600.png]

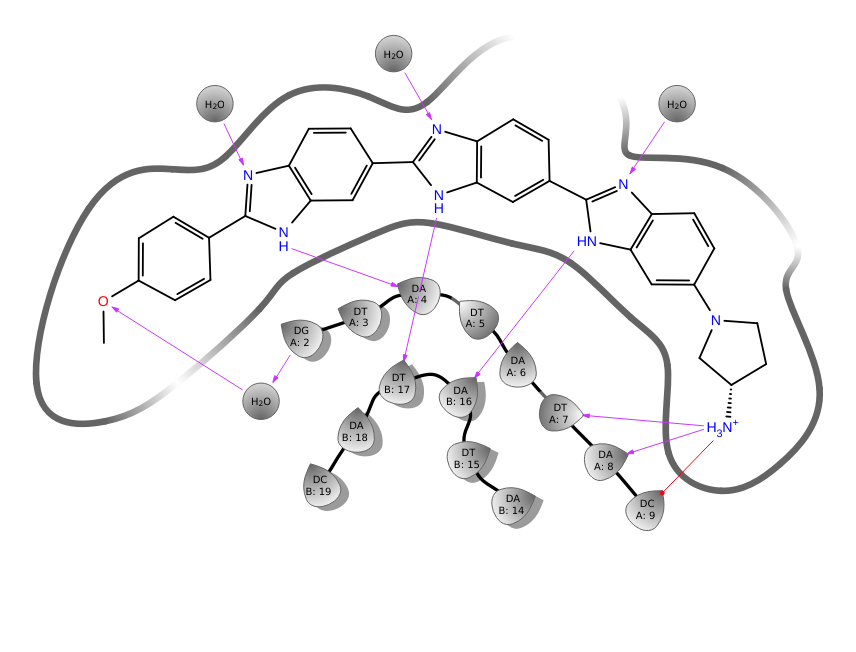

Supplement: S5 Data — (ZIP) [file pone.0221175.s005.zip › trisbenzimidazole/tribz700.png]

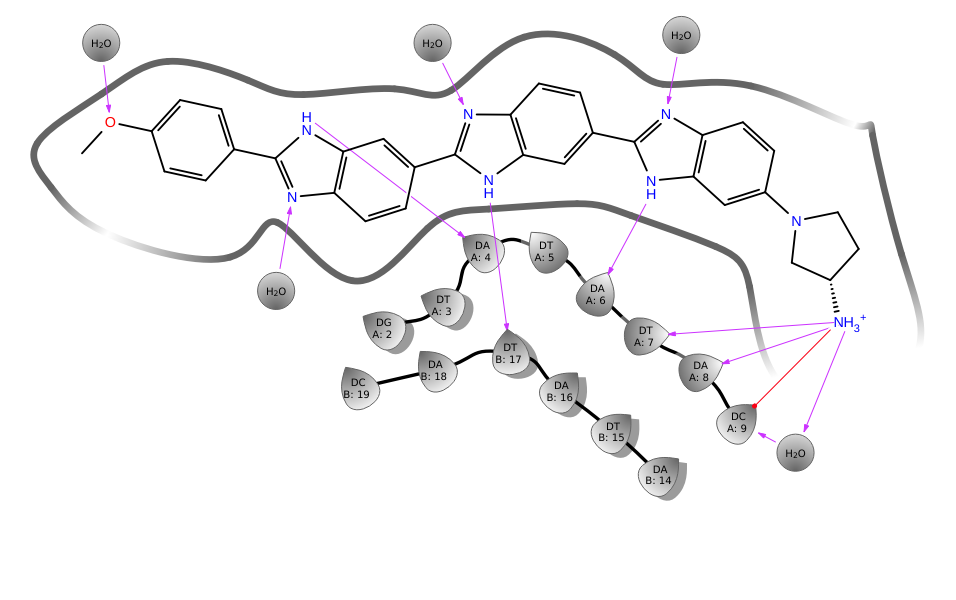

Supplement: S5 Data — (ZIP) [file pone.0221175.s005.zip › trisbenzimidazole/tribz800.png]

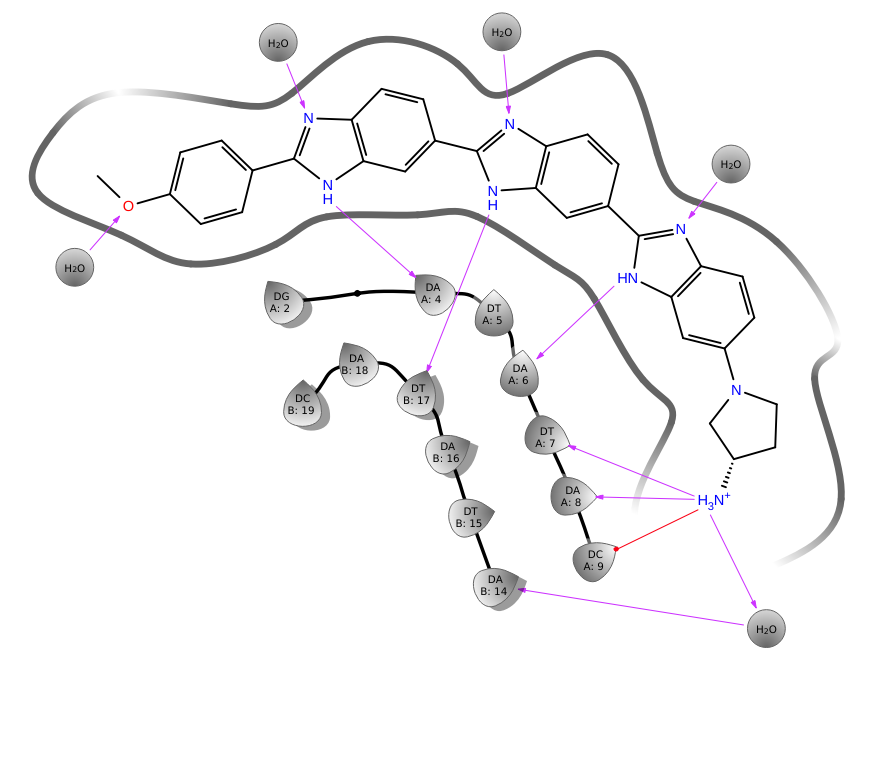

Supplement: S5 Data — (ZIP) [file pone.0221175.s005.zip › trisbenzimidazole/tribz900.png]
